# Investigating the cell of origin and novel molecular targets in Merkel cell carcinoma: a historic misnomer

**DOI:** 10.1002/1878-0261.70107

**Published:** 2025-08-05

**Authors:** Richie Jeremian, Sriraam Sivachandran, Melissa Galati, Brandon Ramchatesingh, Hibo Rijal, Johnny Hanna, Elena Netchiporouk, May Chergui, Margaret Redpath, Samy Abou Setah, Ivan V. Litvinov

**Affiliations:** ^1^ Research Institute of the McGill University Health Centre Montreal Canada; ^2^ Faculty of Medicine University of Toronto Canada; ^3^ Faculty of Medicine Queens University Kingston Canada; ^4^ Faculty of Medicine Laval University Quebec City Canada; ^5^ Department of Pathology McGill University Montreal Canada; ^6^ St. Mary's Research Institute Montreal Canada

**Keywords:** CD19, IgA, Merkel cell carcinoma, PAX5, pre/pro B‐cells, TdT

## Abstract

Merkel cell carcinoma (MCC) is a highly aggressive disease with the poorest prognosis among skin cancers, originally posited to be derived from Merkel cells. Emerging evidence, however, suggests other potential origins for MCC, including hematological lineages. We utilized targeted and multi‐omics approaches to explore gene expression patterns at protein and RNA levels of MCCs. Western blotting, immunofluorescence, and immunohistochemistry were performed using fresh and 92 FFPE samples of primary and metastatic MCC, and two MCC cell lines (MS‐1, HaCaT). RNA sequencing of selected FFPE samples identified differentially expressed genes based on sex and Merkel cell polyomavirus (MCPyV) status. Finally, weighted gene correlation network analysis (WGCNA) and cell type enrichment analyses were employed to determine pathway and cell type enrichment, respectively. MCC patient samples heterogeneously expressed B‐cell and neuroendocrine markers and novel molecular targets including BCMA, CD10, CD93, PAX5, TdT, IgA, and CD19. Transcriptome analysis demonstrated differentially expressed genes based on sex and MCPyV status. MCPyV+ tumors had significant upregulation of genes involved in immune cell function and downregulation of processes related to neuronal activity. WGCNA highlighted enrichment for pathways involved in immune function, including B‐cell differentiation. Cell type enrichment analysis highlighted enrichment for multipotent stem cells, several immune cell types, and keratinocytes. Our findings support previous studies which confirm that MCC is unlikely to be derived from Merkel cells and instead from multiple or divergent cell types, including those of B‐cell lineage. Our work highlights the need for a more personalized approach to diagnosis/characterization and treatment of MCCs, given the documented variability of novel potentially targetable pathways.

## Graphical abstract

This study indicates that Merkel cell carcinoma (MCC) does not originate from Merkel cells, and identifies gene, protein & cellular expression of immune‐linked and neuroendocrine markers in primary and metastatic Merkel cell carcinoma (MCC) tumor samples, linked to Merkel cell polyomavirus (MCPyV) status, with enrichment of B‐cell and other immune cell enrichment in virus‐positive tumors, and nervous tissue enrichment in virus‐negative tumors, collectively suggestive of multiple alternative cellular origins of MCC.

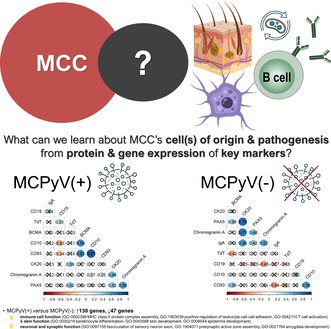

**Abbreviations**
BiTEs: bispecific T‐cell engagers
CAR–T: chimeric antigen receptor T–cell
c–KIT: tyrosine–protein kinase Kit
DAPI: diamidino–2–phenyindle
DEG: differentially expressed gene
DLBCL: diffuse large cell B lymphoma
DMEM: Dulbecco's Modified Eagle Medium
ECL: enhanced chemiluminescence
FBS: fetal bovine serum
FFPE: formalin–fixed paraffin–embedded
GEO: gene expression omnibus
GO:BP: gene ontology biological process
GO:CC: gene ontology cellular component
GTEx: Genotype–Tissue Expression Project
H&E: hematoxylin & eosin
HRP: horseradish peroxidase
IgA: immunoglobulin A
IgG: immunoglobulin G
KEGG: Kyoto Encyclopedia of Genes and Genomes
MCC: Merkel cell carcinoma
MCPyV: Merkel cell polyomavirus
NaOH: sodium hydroxide
OD: optical density
PCA: principal component analysis
Pen–Strep: penicillin–streptomycin
RPMI: Roswell Park Memorial Institute
RRID: research resource identifiers
TCGA: The Cancer Genome Atlas
TRS: target retrieval solution
UV: ultraviolet
WGCNA: weighted gene correlation network analysis

## Introduction

Merkel cell carcinoma (MCC) is a rare and aggressive cutaneous neuroendocrine nonmelanoma skin cancer, known for the poorest patient prognosis of all skin cancers [1, 2]. MCC is associated with several risk factors, namely ultraviolet (UV) exposure, advanced age, and Merkel cell polyomavirus (MCPyV) infection [3, 4, 5]. MCC is more common in immunosuppressed and HIV‐positive individuals, patients with leukemia and lymphoma, and solid organ transplant recipients, who may not be suitable candidates for immunotherapy (*e.g*., heart and liver transplant patients) [6, 7, 8, 9].

Although MCC was initially thought to arise from neuroendocrine skin epidermal cells responsible for hair follicle regulation and mechanical transduction [10, 11], numerous distinctions between MCC and Merkel cells suggest that this may be a historic misnomer. MCC has been described as consisting of small blue (hematoxylin–eosin [H&E] staining) round tumor cells with basophilic nuclei and marginal cytoplasm that grow primarily in the dermis and subcutis, rarely contacting the epidermis [5, 12, 13, 14]. MCC can be classified as trabecular, intermediate, and small cell type, with the small cell subtype being similar to other small cell tumors and resembling sheets of poorly differentiated cells [12, 15, 16]. By contrast, Merkel cells are oval‐shaped postmitotic (have no proliferative ability), localized primarily in the basal layer of the epidermis, and arise from the differentiation of epidermal progenitor cells [17, 18, 19]. MCC and Merkel cells also display distinct structural phenotypes, with the former demonstrating aggregation of plaque‐like filament, while the latter is made up of a more loosely organized filament cytoskeleton [20].

Throughout the published literature, there are several studies that question whether the Merkel cells are the true origin of MCC. Notably, MCC was proposed to express several markers that are absent in Merkel cells, including B‐cell‐specific activator protein (PAX5) and tyrosine‐protein kinase Kit (c‐KIT). This is in keeping with the “*pre*/*pro B‐cell*” hypothesis that proposes that pre/pro‐B‐cells, proliferative cells localized in the dermis and subcutis, are in fact MCC's cell of origin [14]. Indeed, multiple other cells of origin have been proposed for MCC, including multipotent stem, immune, neuronal, and possibly keratinocytes, where sun exposure, immunosuppression, and/or MCPyV may impact/drive diverging MCC phenotypes [21, 22, 23]. Despite the paucity of studies providing alternative evidence regarding MCC's cellular origins, equally few studies have linked MCC with Merkel cells, leaving a knowledge gap in understanding the origin of one of the most fatal cancers. Resolving this matter may uncover novel molecular targets for this malignancy. To address this, we conducted a detailed investigation of B‐cell marker/new molecular target expression in primary and metastatic MCCs, highlighting similarities between this rare skin cancer and B‐cell‐related malignancies.

## Materials and Methods

### Participants and tissue procurement

Initially, two patient volunteers consented to provide tumor lesions to the McGill University Health Center Research Institute (RRID: SCR_014048) blood/skin tissue samples biobank after providing written informed consent, as per the Institutional Review Board‐approved protocol (# 2018‐4128). Patients provided written informed consent and underwent skin punch biopsies of the tumor lesions. Biopsies were immediately placed in cryovials and snap frozen in liquid nitrogen. The study methodologies conformed to the standards set by the Declaration of Helsinki, and all described experiments were undertaken with the understanding and written consent of each subject.

### Tissue procurement

To replicate and expand the data from 2 initial patients, 92 formalin‐fixed paraffin‐embedded (FFPE) patient samples were obtained from the McGill University Health Center Pathology Tissue Biobank (Institutional Review Board Protocol #2022‐8414, RRID: SCR_014048). Blocks available between 2012 and 2022 were included in the study. FFPE samples were processed by the Research Pathology Facility where tissue microarray construction and immunohistochemistry were conducted. Age, sex, and other clinical characteristics are detailed in Table 1 and Table S1. Data on patient sex was available, but data on gender was not recorded in the chart. The analysis was not randomized or blinded.

**Table 1:** Summary of baseline demographics of study patients.

Demographic data	All participants	RNAseq
No. of participants (female)	92 (29)	17 (7)
Age (mean [SD])	76.3 [±10.73]	79.7 [±5.37]
Race	Asian (3); Caucasian (59); Middle Eastern (1); Unknown (29)	Caucasian (12); Unknown (5)
Immunosuppression	Yes (19); No (40); Unknown (33)	Yes (7); No (5); Unknown (5)
MCPyV	Positive (24); Negative (7); Unknown (61)	Positive (5); Negative (2); Unknown (10)
Presence of metastases	Yes (23); No (58); Unknown (11)	Yes (6); No (11); Unknown (11)

### Cell culture

MS‐1 cell line was purchased from Sigma‐Aldrich (09111802, St. Louis, MO, USA, RRID: CVCL_E995) and cells were grown in suspension (unlike most solid tumor cell lines that adhere to the tissue culture plate surface) corresponding to the manufacturer's protocol. Culture medium consisted of Roswell Park Memorial Institute (RPMI) 1640, 10% L‐glutamine, 10% penicillin–streptomycin (Pen‐Strep), and 20% fetal bovine serum (FBS). HaCaT cells were obtained from Dr. Anie Philip's laboratory at the Montreal General Hospital [24], and were cultured corresponding to the manufacturer's protocol. Culture medium consisted of Dulbecco's Modified Eagle Medium (DMEM), 10% Pen‐Strep, and 10% FBS. Cells were seeded at 3 × 10^5^ cells·mL^−1^ in 5% CO_2_ in an incubator at 37 °C. All culture medium materials were purchased from ThermoFisher Scientific (Waltham, MA, USA).

Cell line authentication was performed by ATCC within the past 3 years using Short Tandem Repeat profiling, as a part of their routine authentication and quality control testing of cell line distribution lots. Cells were maintained for a maximum of 25 passages. Cells were tested every 7–10 passages for mycoplasma contamination.

### Western blot

Protein lysates from two patient samples, as well as from MS‐1 (Sigma‐Aldrich 09111802, RRID: CVCL_E995) and HaCaT (Sigma‐Aldrich) cell lines were analyzed using the Bio‐Rad Laboratories Bradford Protein Assay, with protein lysates loaded into each lane of the Mini Protean TGX 50 mL gel. Upon completion, the gel was transferred to the Trans‐Blot Turbo membrane (Bio‐Rad Laboratories, Saint‐Laurent, QC, Canada). Western blotting was conducted using rabbit monoclonal PAX5 (ab109443, RRID: AB_10862070), TdT (ab155082), IgA (ab214003, RRID: 214003), CD19 (ab134114, RRID: AB_2801636), and CK20 (ab76126, RRID: AB_1310117), purchased from Abcam (Waltham, MA, USA). Rabbit polyclonal B‐actin (PA1‐183, RRID: AB_2539914) was purchased from ThermoFischer Scientific. Rabbit IgG, horseradish peroxidase (HRP)‐linked secondary antibody (7074, RRID: AB_2099233) was purchased from Cell Signaling Technologies (Danvers, MA, USA). Enhanced chemiluminescence (ECL) was used to detect bound protein on the Trans‐blot membrane, which was analyzed using the ChemiDoc Imaging system (Bio‐Rad Laboratories, RRID: SCR_019037).

### Immunofluorescence

MS‐1 cells were adhered onto micro cover slips at the bottom of a 6‐well plate (Avantor, VWR International, Randor, PA, USA) as previously described [25]. Cell pellet formed after centrifugation was resuspended at 1 × 10^6^ cells·mL^−1^ in Dulbecco's Phosphate Buffered Saline (PBS, ThermoFischer Scientific). Upon completion of cell adhesion, cells were fixed and permeabilized with formalin (VWR International) and Triton (Bio‐Rad Laboratories), respectively. Rabbit monoclonal PAX5 (ab109443, RRID: AB_10862070), IgA (ab214003, RRID: 214003), and CD19 (ab134114, AB_2801636) were the primary antibodies (Abcam). Rabbit IgG Alexa Fluor 488 was used as the secondary antibody (4412, Cell Signaling Technology, RRID: AB_1904025). Diamidino‐2‐phenylindole (DAPI) staining was used as a positive control. LumaScope 720 was used for slide imaging, and LumaScope 720 was used for slide analysis (Etaluma, Carlsbad, CA, USA).

### Immunohistochemistry

Ninety‐two FFPE patient samples were used to create the tissue microarray (TMA). Sectioned slides from the TMA were stained with anti‐human primary antibodies purchased from Agilent (Santa Clara, CA, USA). Primary antibodies included mouse anti‐PAX5 (GA650, RRID: AB_2335715), rabbit anti‐TdT (IR093, RRID: AB_2094454), rabbit anti‐IgA (IR510), mouse anti‐CD19 (IR656), mouse anti‐CK20 (GA777), and mouse anti‐Chromogranin A (M0869). Sectioned slides from the TMA were also stained with various rabbit anti‐CD19 (ab134114) antibody concentrations (1 : 100, 1 : 200, 1 : 300). The EnVision FLEX Target Retrieval Solution (TRS), 50× Tris/EDTA pH 9 was used for antigen retrieval for 30 min. Slides were then stained with the primary antibodies for 20 min. Upon completion, the EnVision FLEX Substrate Working Solution was used to stain the slides. Slides were counterstained with hematoxylin (Agilent) and subsequently mounted. A second round of staining was conducted on sectioned slides with anti‐human primary antibodies, including anti‐rabbit BCMA (88183, Cell Signaling Technology), anti‐rabbit BOB1 (294R‐17, Sigma‐Aldrich), anti‐rabbit CD10 (PA5‐29354, Invitrogen, Waltham, MA, USA), anti‐rabbit CD22 (98 035, Cell Signaling Technology), anti‐rabbit CD38 (PA5‐84111, Invitrogen), anti‐rat CD45R (11–040282, Invitrogen), and anti‐rabbit CD93 (PA5‐50583, Invitrogen). Immunohistochemistry was performed using the DISCOVERY Ultra instrument (Ventana Medical Systems, Oro Valley, AZ, USA, RRID: SCR_021254). After antigen retrieval treatment with Tris‐EDTA for 32 min (BOB1, CD10) or Citrate buffer for 64 min (BCMA, CD22, CD38, CD45R, CD93), TMA sections were incubated for 24 (Tris‐EDTA) or 60 (Citrate) minutes at 37 degrees Celsius. Secondary antibody incubation was carried out using the OmniMap anti‐Rb HRP (760‐4311, Roche, Basel, Switzerland RRID: AB_2811043) at room temperature for 20 min, followed by standard processing using the UltraView Universal Alkaline Phosphatase Red Detection Kit. Slides were then counterstained with the hematoxylin, dehydrated, cleared and cover slipped. All slide images were captured using the Aperio AT Turbo Slide Scanner and analyzed using Aperio ImageScope (Leica Biosystems, Waltham, MA, USA, RRID:SCR_020993). Differences in the degree of immunofluorescence (measured as optical density [OD]) between primary and metastatic MCC samples were assessed for significance using the Wilcoxon test, while marker co‐expression analysis was conducted using Spearman correlation. All plots were generated using rstudio, Boston, MA, USA 2023.12.10 Build 369 (R 4.3.2, RRID: SCR_000432).

### RNA sequencing

Total RNA was quantified for 17 FFPE‐preserved MCC samples, and its integrity was assessed on a PerkinElmer LabChip GXII (Waltham, MA, USA, RRID: SCR_018593). rRNA was depleted from 125 ng of total RNA using the QIAseq FastSelect platform (QIAGEN, Hilden, Germany). cDNA synthesis was achieved with the NEBNext RNA First Strand Synthesis and NEBNext Ultra Directional RNA Second Strand Synthesis Modules. The remaining steps of library preparation were carried out using the NEBNext Ultra II DNA Library Prep Kit for Illumina (New England BioLabs, MA, USA). Adapters and PCR primers were purchased from New England BioLabs. Libraries were quantified using the KAPA Library Quantification Complete Kit (Kapa Biosystems, Wilmington, MA, USA). Average size fragment was determined using a LabChip GXII (PerkinElmer) instrument. The libraries were normalized and pooled and then denatured in 0.05 N NaOH and neutralized using HT1 buffer. The pool was loaded at 175pM on the Illumina NovaSeq 6000 Sequencer System using the Xp protocol per the manufacturer's recommendations (RRID: SCR_016387). The run was performed for 2 × 100 cycles (paired‐end mode). A phiX library was used as a control and mixed with libraries at a 1% level. Base calling was performed using Real Time Analysis (v3.4.4, RRID: SCR_014332). The bcl2fastq2 (v2.20, RRID: SCR_015058) platform was then used to demultiplex samples and generate FASTQ reads, which were subsequently aligned to the hg19 reference genome (Illumina, San Diego, CA, USA). The above workflow was implemented as previously described [26]. Detailed participant and sample data are highlighted in Table S2A.

### Transcriptomic analysis

Quality control and subsequent analyses were performed using RStudio 2023.12.10 Build 369 (R 4.3.2). The *Rsubread* R package (SCR_016945) was used to extract raw read counts for each gene (25 121 total genes profiled) [27]. The *WGCNA* R package (RRID:SCR_003302) was used to remove outlier genes (*n* = 581) [28]. The *DESeq2* R package (RRID:SCR_015687) was used to remove all genes with total counts < 15 in more than 75% of samples, after which 17 531 genes remained. *DESeq2* was then used to normalize raw count data via variance stabilizing transformation, perform principal component analysis (PCA) and assess for differentially expressed genes (DEGs) between MCC samples based on sex, immunosuppressed status, MCPyV status and tumor type (primary/metastatic) [29]. DEGs were filtered based on an adjusted (False Discovery Rate or FDR) significance value of < 0.01 and log2FoldChange of > 2 and < −2. Enrichment analysis on significant DEGs was performed using the Gene Ontology Biological Process (GO:BP) and Cellular Component (GO:CC) databases [30].

The *WGCNA* R package was then used to construct a signed gene expression network of co‐expressed genes and determine soft thresholding power for the model (soft threshold 18, chosen as being above signed *R* ^2^ threshold above 0.8 while maximizing mean connectivity; Fig. S1). Weighted gene co‐expression network modules were annotated to identify gene symbols using the SynGO ID Conversion Tool (RRID:SCR_017330) [31]. Biological process pathway enrichment analysis using the Kyoto Encyclopedia of Genes and Genomes (KEGG, RRID: SCR_012773) and Gene Ontology (GO, RRID: SCR_002811) databases was performed for each module of genes using the *ShinyGO* platform version 0.77 (RRID: SCR_019213) [30, 32, 33]. KEGG pathways containing expressed genes were visualized using the *Pathview* platform (RRID: SCR_002732) and summary plots of KEGG and GO enrichment were plotted using the *SRplot* platform (RRID: SCR_025904) [34, 35].

Cell type enrichment analysis was performed using the *xCell* algorithm (RRID: SCR_026446) that can identify transcriptional signatures for 64 cell types [36]. Differences in mean cell type enrichment were compared between our MCC cohort (*n* = 17) and cohorts of diffuse large B‐cell lymphoma (DLBCL, *n* = 48) from The Cancer Genome Atlas (TCGA, RRID: SCR_003193) and healthy skin from sites unexposed to sunlight (*n* = 603) from the Genotype‐Tissue Expression Project (GTEx, v8 release, RRID: SCR_013042), and assessed for significance using the Kruskal–Wallis test, adjusted for multiple comparisons using a *post hoc* Dunn's test [37, 38]. All figures were plotted using RStudio 2023.12.10 Build 369 (R 4.3.2).

## Results

We undertook a detailed, multi‐platform approach to investigate the expression of immune/hematologic phenotype (with the focus on B‐cell related markers) in MCC. Our clinical cohort of MCC patients (sex, age, and other clinical characteristics) is summarized in Table 1 (described in detail in Table S1).

### Western blot and immunofluorescence of neuroendocrine and B‐cell‐related markers in MCC

**Fig. 1:**
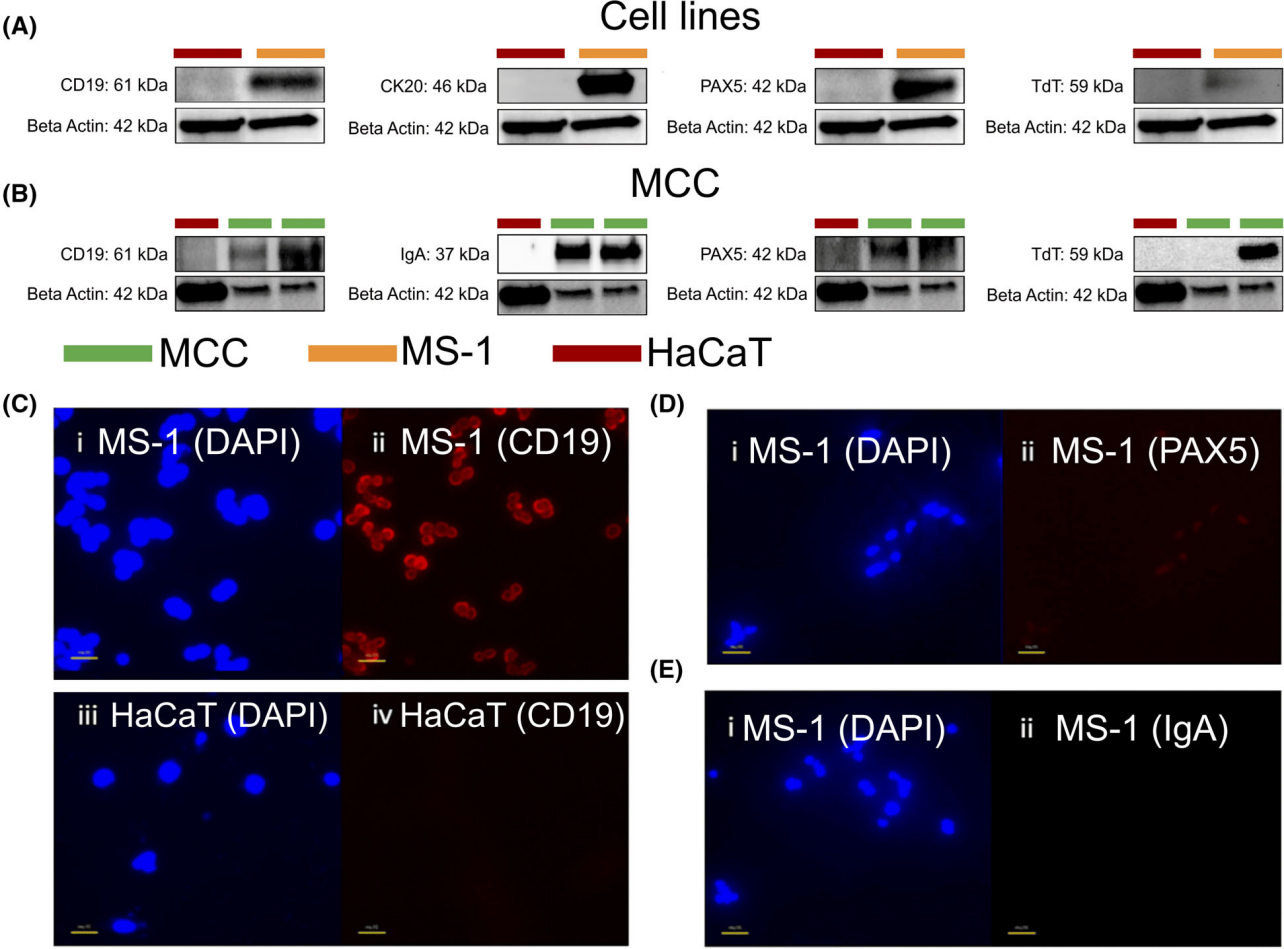
Overview of expression of conventional Merkel cell carcinoma (MCC) and B‐cell‐related markers in two MCC patients (*n* = 2 per tested marker), as well as two cell lines (HaCaT and MS‐1, *n* = 1 per tested marker) via western blot (A, B) and in MS‐1 and HaCaT cells via immunofluorescence (C–E). Western blotting identified moderate‐to‐strong expression of these markers in at least one MCC sample and in MS‐1, but not HaCaT cells. Immunofluorescence of MS‐1 cells revealed strong expression of CD19 (C‐ii), weak expression of PAX5 (D‐ii) and no expression of IgA (E‐ii), and no expression of any marker in HaCaT cells. Western blot samples are labeled using colored bars to indicate MCC (green), HaCaT cells (red) and MS‐1 cell line (orange), with ß‐Actin acting as a positive control. Immunofluorescence findings are presented at 40× magnification with 4′,6‐diamidino‐2‐phenylindole (DAPI, blue) and marker‐specific antibody staining (red). Expression studies were carried out under the same exposure and gain settings.

We began our exploration by investigating the expression of MCC (CK20 and Chromogranin A) and B‐cell markers (PAX5, IgA, TdT, CD19) in two of our freshly obtained MCC samples, via western blotting, using MS‐1 and HaCaT cell lines as positive and negative controls, respectively. Expression of both CK20 and Chromogranin A was not evaluated in patient samples as they were already confirmed to be positive from pathology reports. CK20 was strongly expressed in MS‐1 cells but was not expressed in HaCaT cells. PAX5 was moderately expressed in both patient samples and was strongly expressed in MS‐1, but not HaCaT cells. TdT was strongly expressed in one of two patient samples and in MS‐1 cells, but not in HaCaT cells. IgA was strongly expressed in both patient samples, but not in HaCaT cells. CD19 was weakly expressed in one patient sample and strongly expressed in the other patient sample, as well as in MS‐1, but not HaCaT cells (Fig. 1A,B). Expression of B‐cell markers (PAX5, IgA, CD19) was also assessed using immunofluorescence in MS‐1 and HaCaT cell lines. Weak PAX5 expression and no IgA expression were observed in both cell lines, while CD19 was expressed in MS‐1, but not HaCaT cells (Fig. 1C–E). Expression studies were carried out under the same exposure and gain settings.

### Immunohistochemistry of neuroendocrine and B‐cell‐related markers in MCC

**Fig. 2:**
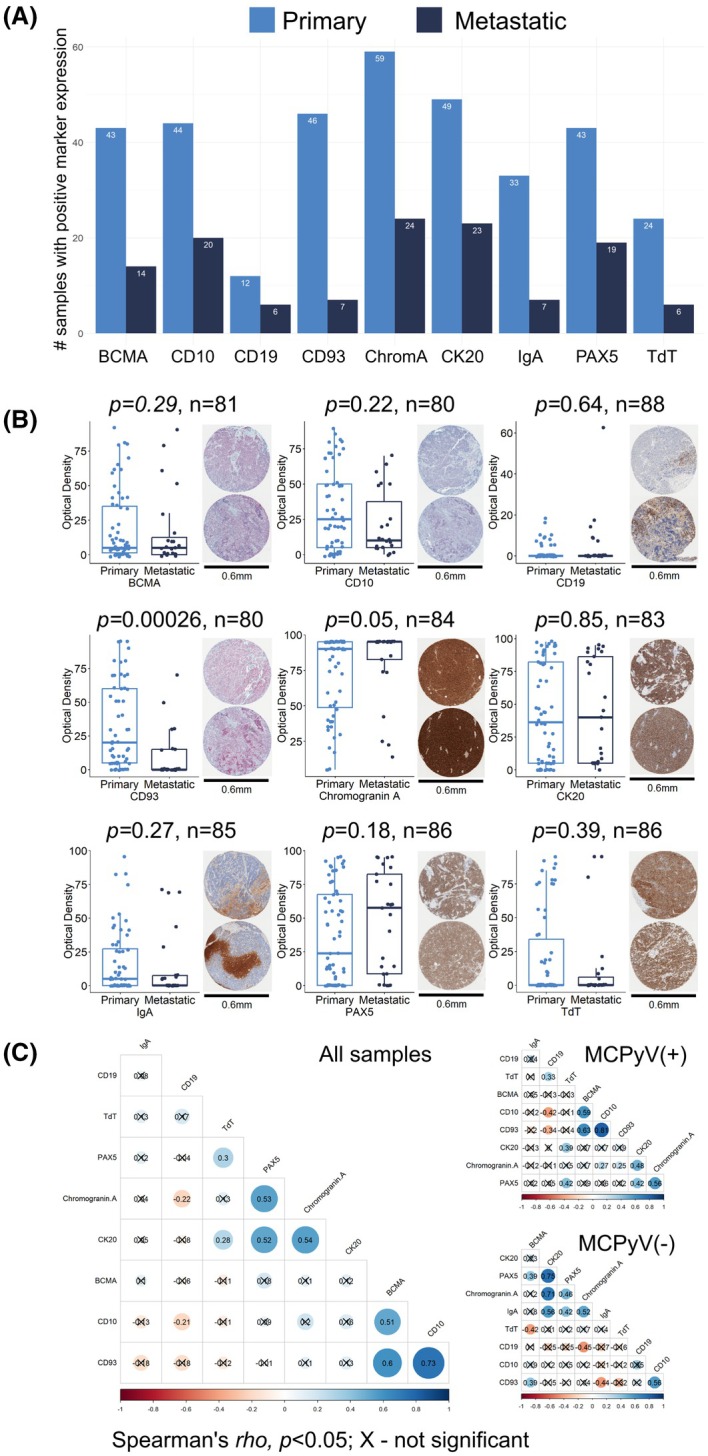
Immunohistochemical analysis of 92 Merkel cell carcinoma (MCC) samples reveals significant interindividual expression for numerous neuroendocrine/B‐cell‐related markers (BCMA, CD10, CD19, CD93, Chromogranin A, CK20, IgA, PAX5, TdT). (A) Overview of primary and metastatic MCC samples with positive marker expression shows abundant expression (> 50% samples) of Chromogranin A, followed by CK20, CD10, PAX5, BCMA and CD93, moderate expression (25–50% samples) of IgA and TdT, and modest expression (< 25% samples) of CD19. (B) Breakdown of individual marker expression (measured as optical density [OD]) by tumor type reveals significantly higher expression of CD93 and lower expression of Chromogranin A in primary *versus* metastatic samples (mean OD, Wilcoxon test, *P* < 0.05, *n* – number of tested samples); bars indicate standard error of the mean. Representative positive samples are shown for each marker from the primary (top) and metastatic (bottom) group (visualized at 15× magnification). (C) Co‐expression analysis of immunohistochemical markers from all MCC samples (left) reveals two distinct networks of moderate‐to‐strong correlation of B‐cell (BCMA, CD10 and CD93) and neuroendocrine with B‐cell (Chromogranin A, CK20 and PAX5) markers (Spearman's *rho* > 0.5). This trend was also observed in sub‐analyses of primary (top right) and metastatic (bottom right) samples.

We next sought to validate the above findings by expanding our investigation of neuroendocrine and B‐cell marker expression in MCC FFPE samples via immunohistochemistry. The complete tissue microarray contained 2 control samples (tonsil and colon) and 92 MCC FFPE patient samples (Figs S2–S10). We observed significant inter‐individual variation in the expression of nine of 13 tested markers (BCMA, CD10, CD19, CD93, Chromogranin A, CK20, IgA, PAX5, TdT) across our cohort, with the remaining four markers showing no expression (BOB1, CD22, CD38, CD45R). The most frequently expressed markers were Chromogranin A (expressed in 91.3% of all tested samples), CK20 (80.4%), CD10 (69.6%), PAX5 (68.5%), BCMA (62.0%) and CD93 (57.6%). The remaining markers were weakly to moderately expressed, including IgA (47.8%), TdT (33.7%) and CD19 (19.6%). By contrast, the control samples were negative for CK20, Chromogranin A, TdT, and IgA expression and positive for BCMA, CD10, CD19, CD93, and PAX5 in the tonsil but not colon control sample (Fig. 2A). Although mean expression of each marker was comparable between tumor types, we observed that primary *versus* metastatic samples exhibited higher CD93 (*P* = 2.6 × 10^−4^) and lower Chromogranin A expression (*P* = 0.05), with no significant differences observed for other markers (Fig. 2B). Marker association analyses of all samples revealed two distinct co‐expression groups of neuroendocrine/B‐cell (TdT, PAX5, Chromogranin A, CK20: *ρ* = 0.28–0.54, *P* < 0.05) and B‐cell (BCMA, CD10, CD93: *ρ* = 0.51–0.73, *P* < 0.05) markers. A similar trend was observed in subgroup analysis of primary tumor samples with neuroendocrine/B‐cell (Chromogranin A, CK20, PAX5, TdT: *ρ* = 0.39–0.56, *P* < 0.05) and B‐cell (BCMA, CD10, CD93: *ρ* = 0.59–0.81, *P* < 0.05) markers showing distinct clusters of expression. By contrast, metastatic MCC tumors predominantly showed co‐expression of B‐cell and neuroendocrine markers (Chromogranin A, CK20, PAX5, IgA: *ρ* = 0.42–0.75, *P* < 0.05), with only moderate co‐expression of BCMA, CD10, and CD93 (*ρ* = 0.39–0.56, *P* < 0.05) (Fig. 2C).

### Gene expression of neuroendocrine and B‐cell markers in MCC samples

We investigated the expression of neuroendocrine and B‐cell markers on the molecular level, using transcriptomic data from a subset (*n* = 17) of our MCC cohort (Table 2). We investigated the expression of genes corresponding to the above‐tested markers as well as additional B‐cell or targetable genes: *PAX5, TdT [DNTT], IgA [IGHA1], CK20 [KRT20], Chromogranin A [CHGA], CD19, BCMA, CD7, CD10, CD20, CD22, CD34, CD38, CD40, CD45, CD52, CD79a, CD79b, Rag1, Tdif1*, *and VREB1*. Among our initially tested markers, all but TdT (*DNTT*) and *CD19* were expressed in all samples. Among the remaining markers, all but *CD52, CD79b, Rag1*, and *VPREB1* were expressed in all samples (Table 2). Together, protein and RNA expression data support several opportunities for therapeutic targeting in these cancers (summarized in Table S3).

**Table 2:** Expression of key neuroendocrine and B‐cell‐related markers among Merkel cell carcinoma samples in RNAseq data (Expressed) and weighted gene correlation network analysis (WGCNA).

Marker	Gene	Name	ENSEMBL ID	Expressed	WGCNA
PAX5	*PAX5*	Paired box 5	ENST00000358127	Yes	No
TdT	*DNTT*	DNA nucleotidylexotransferase	ENSG00000107447	No	No
IgA	*IGHA1*	Immunoglobulin heavy constant alpha 1	ENSG00000211895	Yes	Yes
CK20	*KRT20*	Keratin 20	ENSG00000171431	Yes	No
CHGA	*CHGA*	Chromogranin A	ENSG00000100604	Yes	No
CD19	*CD19*	CD19	ENSG00000177455	No	No
BCMA	*TNFRSF17*	TNF receptor superfamily member 17	ENSG00000048462	Yes	No
CD7	*CD7*	CD7	ENSG00000173762	Yes	Yes
CD10	*MME*	Membrane metalloendopeptidase	ENSG00000196549	Yes	No
CD20	*MS4A1*	Membrane spanning 4‐domains A1	ENSG00000156738	Yes	Yes
CD22	*CD22*	CD22	ENSG00000012124	Yes	Yes
CD34	*CD34*	CD34	ENSG00000174059	Yes	No
CD38	*CD38*	CD38	ENSG00000004468	Yes	Yes
CD40	*CD40*	CD40	ENSG00000101017	Yes	Yes
CD45	*CD45*	Protein tyrosine phosphatase receptor type C	ENSG00000081237	Yes	No
CD52	*CD52*	CD52	ENSG00000169442	No	No
CD79a	*CD79a*	CD79A	ENSG00000105369	Yes	Yes
CD79b	*CD79b*	CD79b	ENSG00000007312	No	No
Rag1	*Rag1*	Recombination activating 1	ENSG00000166349	No	No
Tdif1	*DNTTIP1*	Deoxynucleotidyltransferase terminal interacting protein 1	ENSG00000101457	Yes	No
VPREB1	*VPREB1*	V‐set pre‐B cell surrogate light chain 1	ENSG00000169575	No	No

### Differential gene expression based on sex and MCPyV status

We then assessed for groupwise differences in our cohort by sex, MCPyV status, immunosuppression status, and tumor type (primary *vs*. metastatic), the former two showing distinct clustering on PCA (Figs S11–S14). We identified 24 genes that were upregulated and 88 that were downregulated in female *versus* male participants, as well as 138 upregulated and 47 downregulated genes in MCPyV positive *versus* negative individuals (Table S2A,F, Figs S15 and S16). There were no significant DEGs based on immunosuppression status or tumor type (Figs S17 and S18).

Gene Ontology analysis of DEGs revealed intriguing differences based on MCPyV positive status, including 48 biological processes and 61 cellular components among upregulated genes, and 357 biological processes and 100 cellular components among downregulated genes (Table S2H–K). Twenty‐eight of 48 biological processes among upregulated genes were related to immune cell function (GO:0002399 MHC class II protein complex assembly, GO:1903039 positive regulation of leukocyte cell–cell adhesion, GO:0042110T cell activation), and to a lesser degree, skin function (GO:0030216 keratinocyte differentiation, GO:0043588 skin development, GO:0008544 epidermis development). By contrast, downregulated genes among MCPyV‐positive individuals were enriched for processes related to broader cell function and development, with a slight predominance of biological processes related to neuronal and synaptic function (GO:0097155 fasciculation of sensory neuron axon, GO:1904071 presynaptic active zone assembly, GO:0021764 amygdala development), corroborated by 38 cellular components related to neuronal and synaptic function (including GO:0098793 presynapse, GO:0097060 synaptic membrane, GO:0043679 axon terminus). These findings highlight significant differences in gene expression and cellular composition in MCC patients on the basis of MCPyV status, suggesting differences in underlying pathophysiology related to patient demographics.

### Weighted gene correlation network analysis of MCC samples

**Fig. 3:**
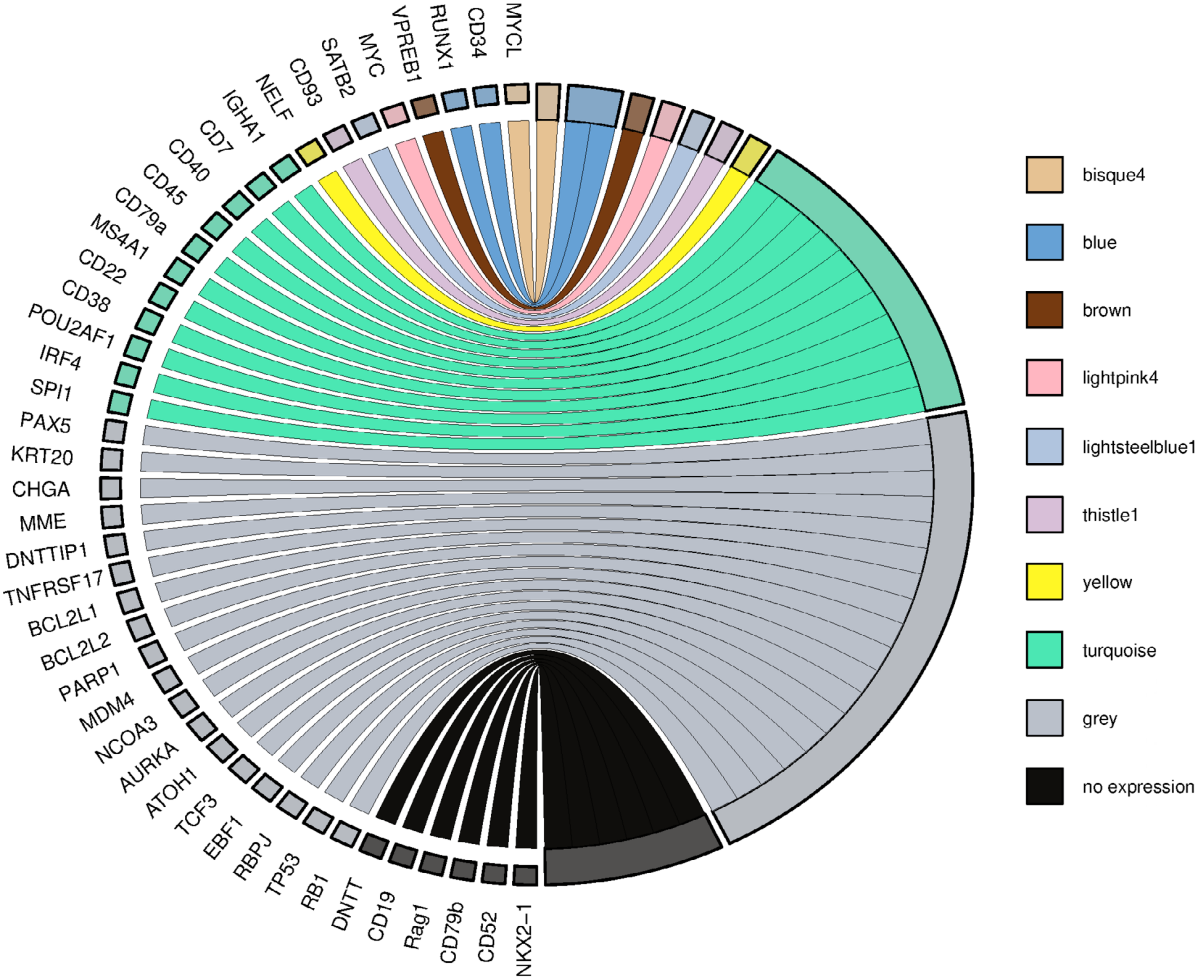
Chord plot of genes corresponding to neuroendocrine and B cell function, as well as those previously found to be expressed in Merkel cell carcinoma (MCC), and their association with modules derived from weighted gene correlation network analysis of 17 MCC transcriptomes. Figure legend highlights module names; the gray cluster represents genes that did not correspond to any modules, while the black cluster represents genes that were not expressed in this cohort.

We next performed weighted gene correlation network analysis (WGCNA) using transcriptomic data from our cohort of MCC patients to identify networks of genes (modules) that are co‐expressed. This analysis yielded 57 co‐expression modules across a total of 17 531 genes (Fig. S19, Table S4A). Notably, one of these modules (turquoise), containing 662 genes, was observed to be distinctly enriched for immune cell‐related, including B‐cell‐related processes, and contained seven genes corresponding to the aforementioned transcriptomic markers (*CD7, CD20 [MS4A1], CD22, CD38, CD40, CD79a, IgA [IGHA1], IRF4, POU2AF1 and SPI1*). Another seven modules collectively contained genes previously associated with hematopoiesis and B cell function (CD34, *RUNX1* and *VPREB1*), MCC (*SATB2*), and carcinogenesis (*MYC, MYCL, NELF, CD93*), summarized in Fig. 3.

**Fig. 4:**
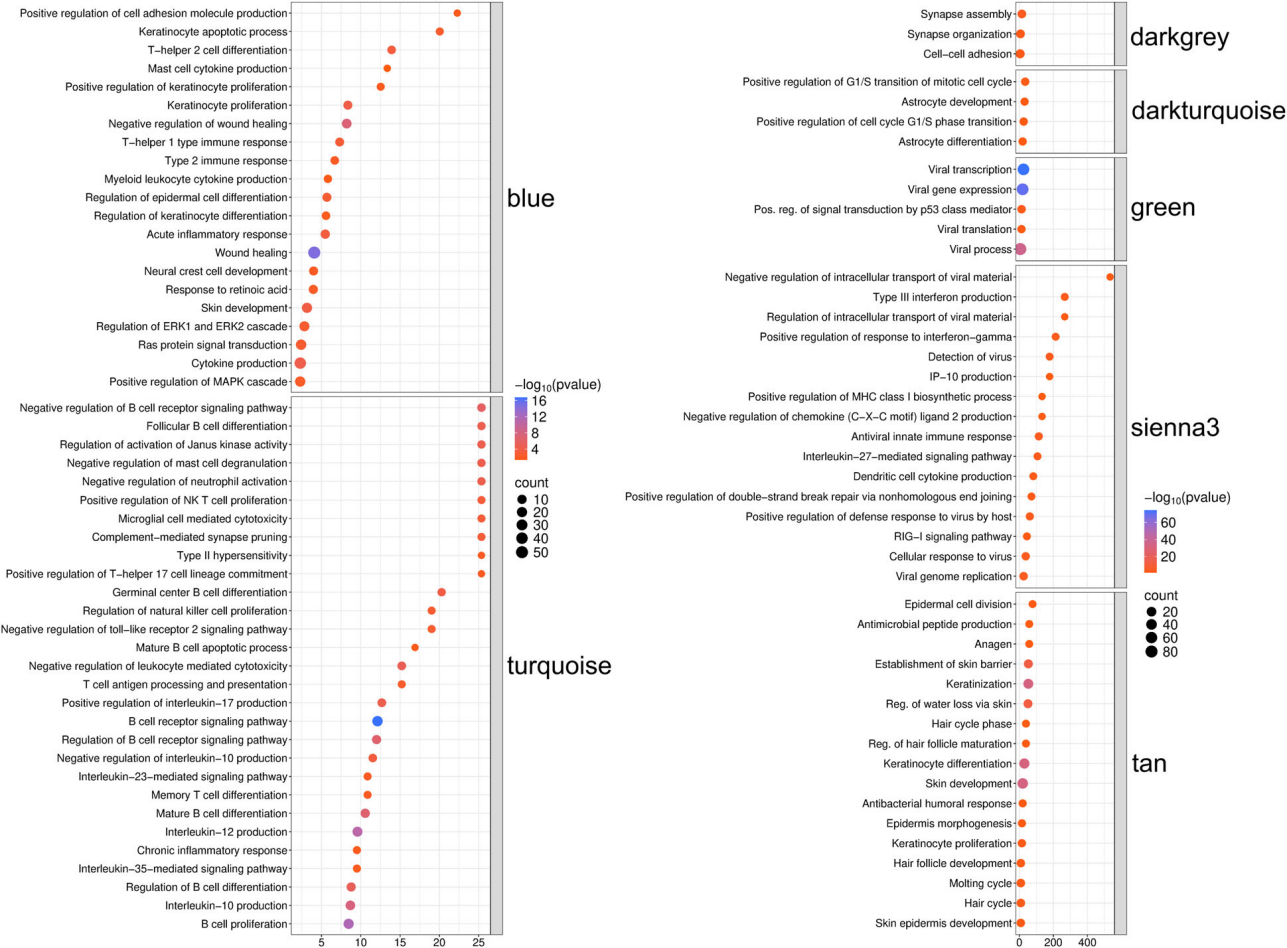
Enrichment analysis (Gene Ontology: Biological Process) of expressed genes in seven modules (blue, turquoise, dark gray, dark turquoise, green, sienna3 and tan) derived from weighted gene correlation network analysis of 17 Merkel cell carcinoma (MCC) transcriptomes reveals module‐specific enrichment for pathways associated with skin cell function and corresponding immune signaling (blue and tan), B‐cell development and differentiation, activation, signaling, and apoptosis (turquoise), brain cell function (dark gray and dark turquoise), viral processes (green and sienna3). Fold enrichment is shown for each pathway (*X*‐axis) with color grading representing the significance of enrichment (−log_10_[FDR *P*‐value]).

**Fig. 5:**
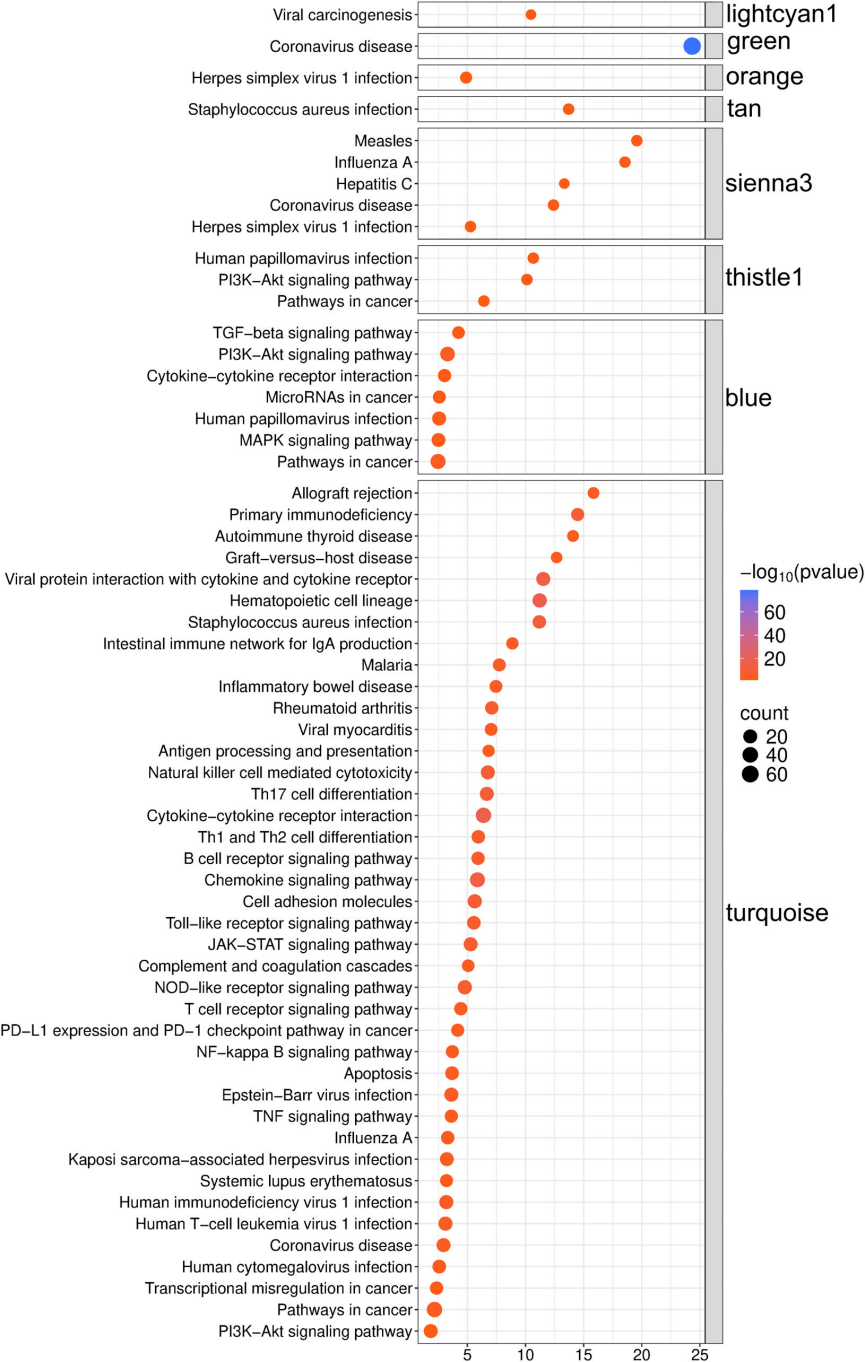
Overview of enrichment analysis (Kyoto Encyclopedia of Genes and Genomes) of expressed genes in eight modules (lightcyan1, green, orange, tan, sienna3, thistle1, blue and turquoise) derived from weighted gene correlation network analysis of 17 Merkel cell carcinoma (MCC) transcriptomes reveals a predominance of pathways associated with immune cell function and signaling, infectious and inflammatory disease, and malignancy. Pathways are sorted based on fold enrichment (*X*‐axis), with circle size proportional to the number of positively expressed genes in our cohort corresponding to each pathway, and color grading representing the significance of enrichment (−log_10_[FDR *P*‐value]).

Downstream GO (Fig. 4) and KEGG (Fig. 5) pathway enrichment analysis of genes in each module identified 2533 GO Biological Processes and 126 KEGG pathways across all modules (Table S4B,C), highlighting that co‐expressed genes in our MCC cohort correspond to modules with distinct functions. Per GO pathway analysis, these include immune cell function and signaling (turquoise and sienna3), skin cell proliferation and function (blue and tan), brain cell‐related function (dark gray and dark turquoise), and viral infection and corresponding processes (green and sienna3). Per KEGG pathway analysis, these include viral infection and cancer‐related processes (lightcyan1, green, orange, tan, thistle1 and blue), as well as infectious diseases, immune signaling, and immunodeficiency, and cancer (turquoise). Collectively, these findings demonstrate the broad spectrum of gene expression in MCC, highlighting the role of immune (B cell predominant) and viral processes that mediate carcinogenesis.

### Cell enrichment analysis of MCC samples

We compared the enrichment of cell types between transcriptome data from MCC, DLBCL, and healthy skin using an algorithm that provides enrichment scores for 64 cell types. Among our cohort, the top 10 most enriched cell types were multipotent progenitor cells (mean enrichment 0.32, SD 0.18), M2 macrophages (0.28, 0.09), natural killer T cells (0.23, 0.11), mesenchymal stem cells (0.23, 0.14), epithelial cells (0.21, 0.13), keratinocytes (0.18, 0.12), class‐switched memory B‐cells (0.12, 0.08), osteoblasts (0.11, 0.11), granulocyte‐monocyte progenitor cells (0.11, 0.09), and common lymphoid progenitor cells (0.09, 0.06).

**Fig. 6:**
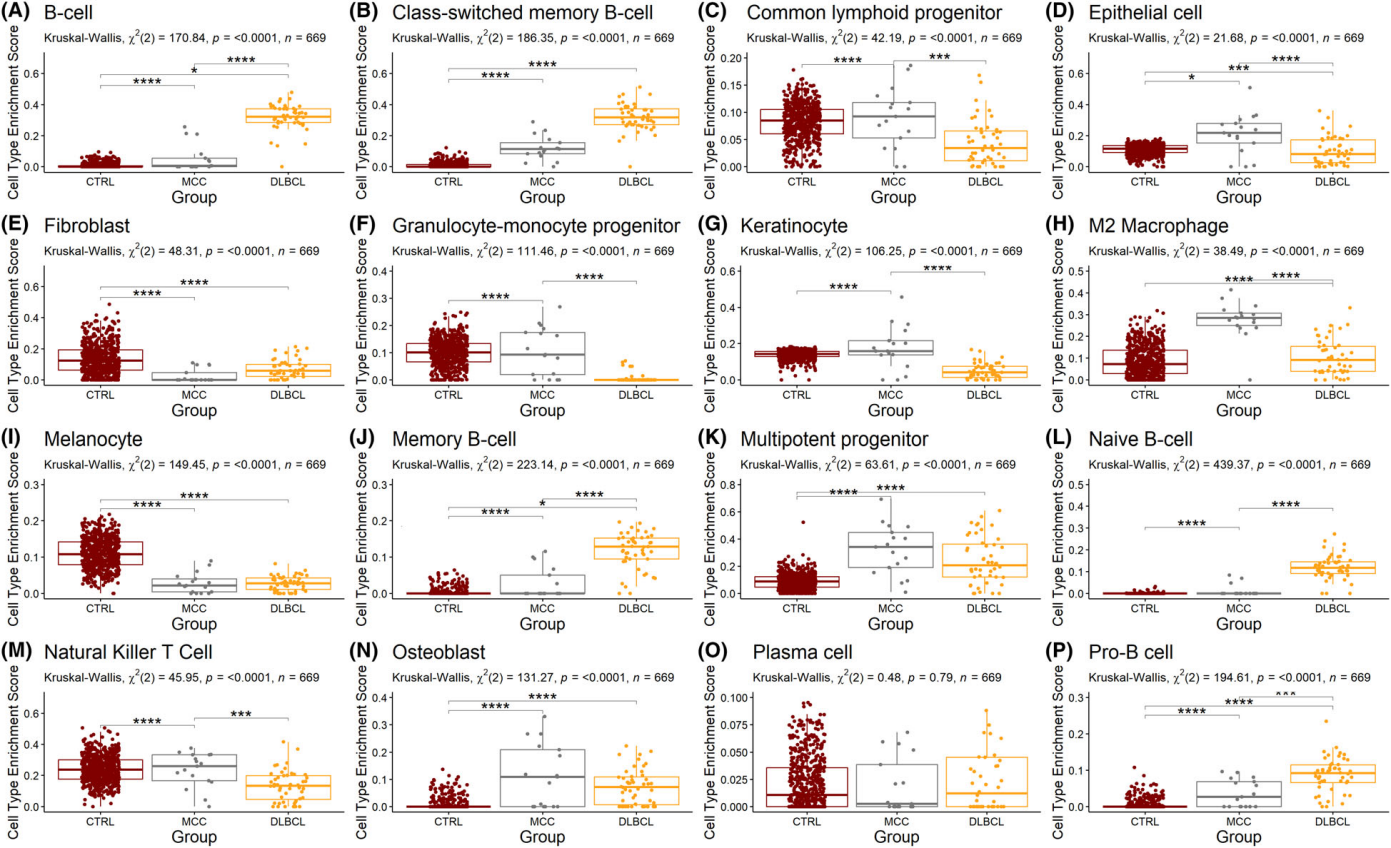
Overview of cell type enrichment analysis reveals significant enrichment differences between 17 Merkel cell carcinoma (MCC) transcriptomes and two publicly available cohorts of diffuse large B‐cell lymphoma (DLBCL, *n* = 48; *TCGA‐DLBC*), and healthy skin (CTRL, *n* = 603; sunlight‐naïve healthy skin, *GTEx v8*). Notably, MCC shows a diverse enrichment phenotype encompassing B‐cell subtype‐, immune‐, progenitor and skin‐related (epithelial cell, fibroblast, melanocyte) cells. An intermediate enrichment phenotype is seen between DLBCL (abundant enrichment) and CTRL (modest enrichment) for five B‐cell subtypes (A [B‐cells], B [Class‐switched memory B‐cells], J [Memory B‐cells], L [Naïve B‐cells], P [Pro B‐cells]). MCC samples also showed significantly more enrichment of two progenitor cell types compared to DLBCL and CTRL (C [Common lymphoid progenitors], K [Multipotent progenitors]). Among non‐immune and progenitor cell types, epithelial cell and keratinocyte enrichment was greatest in MCC compared to other groups (*P* < 0.0001), while fibroblast and melanocyte enrichment was greatest in CTRL, with no differences between MCC and DLBCL (*P* > 0.05). Cell enrichment scores derived from the *xCell* algorithm based on the 64‐cell signature and assessed for inter‐group significance (difference of mean cell enrichment score) using the Kruskal–Wallis test followed by the *post hoc* Dunn's test. * – 0.05 *P* < *** – 0.001 *P* < **** – *P* < 0.0001. Bars indicate standard error of the mean. Cell type enrichment analysis included the following cell types: (A) B‐cells, (B) Class‐switched memory B‐cells, (C) Common lymphoid progenitors, (D) Epithelial cells, (E) Fibroblasts, (F) Granulocyte‐monocyte progenitors, (G) Keratinocytes, (H) M2 macrophages, (I) Melanocytes, (J) Memory B‐cells, (K) Multipotent progenitors, (L) Naïve B‐cells, (M) Nature killer T cells, (N) Osteoblasts, (O) Plasma cells, (P) Pro‐B cells.

Among cell types of B‐cell lineage, we observed significant differential enrichment signals among B‐cells, class‐switched memory B‐cells, memory B‐cells, naïve B‐cells, and pro‐B‐cells, with DLBCL showing the greatest enrichment, and MCC being intermediately enriched between DLBCL and healthy skin (*P* < 0.0001). Among progenitor cells, MCC showed significantly greater enrichment than the other two groups for common lymphoid progenitor cells and granulocyte‐monocyte progenitor cells, while the enrichment of multipotent progenitor cells was significantly greater in MCC and DLBCL compared to healthy skin (*P* < 0.0001). Among other immune cell types, MCC exhibited greater enrichment of M2 macrophages and natural killer T‐cells compared to the other two groups (*P* < 0.0001), while no significant groupwise differences in plasma cell enrichment were observed. We also observed significant differences in the enrichment of non‐immune cell types. Interestingly, epithelial cell and keratinocyte enrichment was significantly greater in MCC samples (*P* < 0.0001). By contrast, fibroblast and melanocyte enrichment was significantly greater in healthy skin compared to the two malignant groups, with no significant enrichment differences between MCC and DLBCL (*P* > 0.05). Finally, osteoblast enrichment was significantly greater in both MCC and DLBCL *versus* healthy skin (*P* < 0.0001). Although *xCell* and comparable algorithms lack a Merkel cell signature, these findings demonstrate that MCC tumors contain enrichment signals for mature immune cells, progenitor cells, keratinocytes, and other non‐immune cells that collectively overlap with enrichment signals from both DLBCL and healthy skin (Fig. 6A–P).

## Discussion

MCCs are highly aggressive tumors known for their poor prognosis. As such, determining potential targetable markers and pathways is an important priority. Essential to determining treatment targets is an understanding of the MCC cell of origin. Although MCCs have historically been thought to arise from Merkel cells [14], largely due to the expression of CK20 and CD56 [39], new hypotheses have been proposed in recent years. A recent intriguing hypothesis endorses hematologic/multipotent progenitor cell or pre/pro B‐cells as the MCC cell of origin [40], based on several observations.

Firstly, Merkel cells are found in the basal layer of the epidermis, while MCCs are found in the subcutis and dermis [41]. Merkel cells are post‐mitotic and are found only sparsely in the epidermis; therefore, making them unlikely to be the primary progenitors for MCCs. Moreover, several features of MCC are atypical of solid tumors and more in keeping with hematological malignancies. MCC is one of the most radiosensitive tumors in dermatology, which is more typical of hematologic malignancies than solid tumors. Also unusual is the lack of an established precursor lesion linking normal Merkel cells to MCC, unlike many solid cancers that show clear precancerous stages. Patient‐derived cell lines of MCC grow in suspension, more in keeping with blood cancer cell lines than those from solid tumors. Clinically, MCC presents as violaceous dermal nodules, much like cutaneous lymphomas. Pathologically, these tumors too consist of small blue cells resembling lymphoid malignancies. Nevertheless, in addition to hematologic/B‐cell lineage, it is important to consider other possibilities.

Our study presents a multi‐omics approach that supports several hypotheses suggesting alternative origins for MCCs. We and others report the aberrant expression of B‐cell lineage markers in MCC tumors. These include CD20, a pan‐B‐cell marker, and other proteins typically associated with B‐cell activation and differentiation, such as PAX5 (a B‐cell transcription factor) and BCL‐2 (an anti‐apoptotic protein often expressed in B‐cell malignancies). The expression of PAX5, a transcription factor essential for B‐cell development, has been identified in MCC tumors, further supporting a potential B‐cell lineage. Additionally, BCL‐2 overexpression, commonly observed in B‐cell lymphomas, has been reported in MCC, suggesting similarities in the survival mechanisms between MCC and B‐cell malignancies. At the protein level, we demonstrated that MCC patient‐derived samples expressed both neuroendocrine/MCC (Chromogranin A and CK20) and B‐cell markers (BCMA, CD10, CD19, CD93, PAX5, TdT, and IgA), with certain sample populations showing a preference for co‐expression of markers from one of these two groups (Figs 1 and 2), collectively supporting previously published studies [40]. Our RNA sequencing and weighted gene correlation network analysis of 17 531 genes among MCC samples identified 57 clusters of co‐expressed genes. One of these clusters contained numerous genes involved in immune function, including B‐cell related genes, while pathway enrichment analysis demonstrated the greatest number of genes corresponding to immune function with 26 GO processes found to be B‐cell associated (Figs 3, 4, 5, Tables S2 and S4). Finally, cell enrichment analysis with *xCell* revealed an enrichment for cell types including multipotent progenitor cells, mesenchymal stem cells, epithelial cells, keratinocytes, osteoblasts, and various immune cells including M2 macrophages, natural killer T cells, class‐switched memory B‐cells, granulocyte‐monocyte progenitor cells, and common lymphoid progenitor cells (Fig. 6). These findings further support ongoing research that MCCs may have B‐cell and/or numerous cells of origin or cells transformed at various times throughout their differentiation by the MCPyV.

Critical to the mechanism of carcinogenesis in MCC is the discovery of MCPyV in the majority of MCC cases. MCPyV is a small DNA virus that integrates into the host genome, and its large T‐antigen is thought to drive tumorigenesis by disrupting cell cycle regulation. Viral‐positive MCC tumors exhibit viral oncoproteins that are critical for tumor cell survival. The association between MCPyV and MCC further questions the cell of origin, particularly since polyomaviruses have been linked to lymphoid malignancies in experimental models, while Merkel cells are not easily infected by MCPyV [42]. We investigated DEGs in our sample cohort based on viral status. Interestingly, MCPyV+ tumors downregulated neuronal‐related genes, with more than half of upregulated biological processes relating to immune function and signaling (Table S2F–J), similarly suggesting an immune‐related origin for these MCPyV+ tumors. This data is important particularly in the context of existing studies demonstrating that lymphoid tissues could be infected by this virus [43, 44]. This connection suggests that MCPyV may preferentially infect or transform cells of lymphoid lineage, leading to the development of MCC. This hypothesis is consistent with the observation that MCC often arises in immunosuppressed individuals, who may have impaired control over viral infections and malignant transformation, and supports previous transcriptomic studies profiling gene expression and mutational differences between virus‐positive and virus‐negative tumors [45, 46, 47, 48].

Finally, PCA and enrichment analyses highlighted clustering based on differences between female *and* male patients. Interestingly, samples from female patients demonstrated DEGs including upregulation in processes related to cell structure while downregulated genes were related to immune function (Table S2B–F). This highlights the need for a personalized medicine approach in MCC based on sex and MCPyV status to best assess individual patients and tumors and determine expressed targets.

Although the data presented here are indicative of a predominantly B‐cell lineage as the cell of origin for MCCs, ongoing investigations concomitantly posit several alternatives. Recent studies have supported the possibility that MCCs derive from a primitive pluripotent or multipotent stem cell that has the potential for differentiation along different phenotypes including neuroendocrine, glandular, or squamous cell [22, 49, 50]. Our transcriptome data demonstrate enrichment for multipotent progenitors, epithelial cells, and keratinocytes in sequenced MCC tumors (Fig. 6).

These findings should be viewed in light of several limitations. First, the quality of FFPE samples used for microarrays may have been variable given their collection dates ranged from 2012 to 2022. Thus, the quality of the samples may have affected the possible expression of specific B‐cell markers. However, results obtained from western blot analysis and RNA sequencing support these findings. Another possible limitation is the small sample size of patient samples used for western blot analysis and subsequent tumor microarray and RNASeq studies. There were only two patients' biopsies readily available to extract protein, as MCCs are exceedingly rare. Our RNA sequencing data provided increased sample size to validate findings at the protein level. Finally, while WGCNA analysis identifies gene clusters whose expression was positively correlated, it cannot determine the level of gene expression.

## Conclusions

This study supports previous findings that suggest multiple alternative cells of origin for MCC, with combined results supporting a hematological cell type, possibly of B‐cell lineage. Determining the origins of MCC will require larger cohorts with detailed clinical characteristics and represents a crucial step to the identification of critically needed novel targetable therapeutic markers, given that MCC tumors typically arise in patient populations not suitable for immunotherapy (*e.g*., solid organ transplant recipients). Our findings demonstrating expression of CD19, for example, in some patients could open a new avenue of therapeutic intervention via chimeric antigen receptor (CAR) T‐cell therapies, bispecific T‐cell engagers (BiTEs), or antibody‐drug conjugates (ADCs) that have been approved for multiple B‐cell malignancies [51, 52, 53]. BCMA targeted therapies including CAR‐T and BiTEs have also been approved in multiple myeloma [51, 54, 55]. Finally, CD93 and TdT have been proposed as promising targets in acute myeloid leukemia (AML) and B‐cell acute lymphoblastic leukemia (ALL) respectively [56, 57, 58].

## Authors' contributions

Conceptualization: IVL; Data curation: IVL; Formal analysis: RJ, SS, MG, BR, IVL; Funding acquisition: IVL; Investigation: RJ, SS, MG, HR, EN, MC, MR, SAS, IVL; Methodology: RJ, SS, MG, BR, HR, JH, EN, MC, MR, SAS, IVL; Project administration: IVL; Resources: MG, IVL; Software: RJ, SS, MG, IVL; Supervision: IVL; Validation: RJ, SS, MG; Visualization: RJ, SS, MG, HR, JH, EN, MC, MR, SAS, IVL; Writing—original draft: RJ, SS, MG, IVL; Writing—review & editing: RJ, SS, MG, BR, HR, JH, EN, MC, MR, AS, IVL. All authors have read and agreed to the final published version of the manuscript.

## Conflict of interest

The authors declare no conflict of interest.

## Supporting information


**Fig. S1.** Soft threshold power of signed weighted gene correlation network analysis model of co‐expressed genes in MCC transcriptomes (soft threshold 18 chosen as being above signed R2 threshold above 0.8 while maximizing mean connectivity).
**Fig. S2.** Tissue microarray of BCMA protein expression in MCC FFPE samples and two control samples (top left: tonsil [top], colon [bottom]).
**Fig. S3.** Tissue microarray of CD10 protein expression in MCC FFPE samples and two control samples (top left: tonsil [top], colon [bottom]).
**Fig. S4.** Tissue microarray of CD19 protein expression in MCC FFPE samples and two control samples (top left: tonsil [top], colon [bottom]).
**Fig. S5.** Tissue microarray of CD93 protein expression in MCC FFPE samples and two control samples (top left: tonsil [top], colon [bottom]).
**Fig. S6.** Tissue microarray of Chromogranin A protein expression in MCC FFPE samples and two control samples (top left: tonsil [top], colon [bottom]).
**Fig. S7.** Tissue microarray of CK20 protein expression in MCC FFPE samples and two control samples (top left: tonsil [top], colon [bottom]).
**Fig. S8.** Tissue microarray of IgA protein expression in MCC FFPE samples and two control samples (top left: tonsil [top], colon [bottom]).
**Fig. S9.** Tissue microarray of PAX5 protein expression in MCC FFPE samples and two control samples (top left: tonsil [top], colon [bottom]).
**Fig. S10.** Tissue microarray of TdT protein expression in MCC FFPE samples and two control samples (top left: tonsil [top], colon [bottom]).
**Fig. S11.** Principal component analysis of MCC transcriptomes based on patient sex.
**Fig. S12.** Principal component analysis of MCC transcriptomes based on patient Merkel cell polyoma virus status.
**Fig. S13.** Principal component analysis of MCC transcriptomes based on patient immunosuppressed status.
**Fig. S14.** Principal component analysis of MCC transcriptomes based on patient tumor type.
**Fig. S15.** Minus‐average plot of differential gene expression of MCC transcriptomes based on patient sex.
**Fig. S16.** Minus‐average plot of differential gene expression of MCC transcriptomes based on patient Merkel cell polyoma virus status.
**Fig. S17.** Minus‐average plot of differential gene expression of MCC transcriptomes based on patient immunosuppressed status.
**Fig. S18.** Minus‐average plot of differential gene expression of MCC transcriptomes based on patient tumor type.
**Fig. S19.** Cluster dendrogram of 57 co‐expression modules across 17,531 genes based on weighted gene correlation network model of MCC transcriptomes.
**Fig. S20.** KEGG enrichment analysis of MCC‐expressed gene correlation network (turquoise module) shows expressed genes in hematopoietic stem cell lineage pathway (hsa04640).
**Fig. S21.** KEGG enrichment analysis of MCC‐expressed gene correlation network (turquoise module) shows expressed genes in B‐cell receptor signaling pathway (hsa04662).
**Fig. S22.** KEGG enrichment analysis of MCC‐expressed gene correlation network (turquoise module) shows expressed genes in pathways in cancer pathway (hsa05200).

**Table S1.** Overview of Merkel cell carcinoma patient demographics.

**Table S2.** Overview of participants in the sequenced cohort, transcriptomic findings (differentially expressed genes) and pathway enrichment analysis thereof.

**Table S3.** Summary of potential novel therapeutic targets for Merkel cell carcinoma.

**Table S4.** Overview of weighted gene correlation network analysis modules and pathway enrichment analysis thereof.

## Data Availability

The RNA‐Seq data generated in this study has been deposited in the Gene Expression Omnibus (GEO) database accession number: GSE284793.
