# Supplementary material for: Investigating the cell of origin and novel molecular targets in Merkel cell carcinoma: a historic misnomer
Source: Mol Oncol. 2025 Aug 5;20(2):331–47. doi: 10.1002/1878-0261.70107 (PMC12936429; doi:10.1002/1878-0261.70107)
Supplement: Supplementary file 1 — Fig. S1. Soft threshold power of signed weighted gene correlation network analysis model of co‐expressed genes in MCC transcriptomes (soft threshold 18 chosen as being above signed R2 threshold above 0.8 while maximizing mean connectivity). Fig. S2. Tissue microarray of BCMA protein expression in MCC FFPE samples and two control samples (top left: tonsil [top], colon [bottom]). Fig. S3. Tissue microarray of CD10 protein expression in MCC FFPE samples and two control samples (top left: tonsil [top], colon [bottom]). Fig. S4. Tissue microarray of CD19 protein expression in MCC FFPE samples and two control samples (top left: tonsil [top], colon [bottom]). Fig. S5. Tissue microarray of CD93 protein expression in MCC FFPE samples and two control samples (top left: tonsil [top], colon [bottom]). Fig. S6. Tissue microarray of Chromogranin A protein expression in MCC FFPE samples and two control samples (top left: tonsil [top], colon [bottom]). Fig. S7. Tissue microarray of CK20 protein expression in MCC FFPE samples and two control samples (top left: tonsil [top], colon [bottom]). Fig. S8. Tissue microarray of IgA protein expression in MCC FFPE samples and two control samples (top left: tonsil [top], colon [bottom]). Fig. S9. Tissue microarray of PAX5 protein expression in MCC FFPE samples and two control samples (top left: tonsil [top], colon [bottom]). Fig. S10. Tissue microarray of TdT protein expression in MCC FFPE samples and two control samples (top left: tonsil [top], colon [bottom]). Fig. S11. Principal component analysis of MCC transcriptomes based on patient sex. Fig. S12. Principal component analysis of MCC transcriptomes based on patient Merkel cell polyoma virus status. Fig. S13. Principal component analysis of MCC transcriptomes based on patient immunosuppressed status. Fig. S14. Principal component analysis of MCC transcriptomes based on patient tumor type. Fig. S15. Minus‐average plot of differential gene expression of MCC transcriptomes based on patient se [file MOL2-20-331-s001.pdf]

Supplementary Figures

Supplementary Figure 1

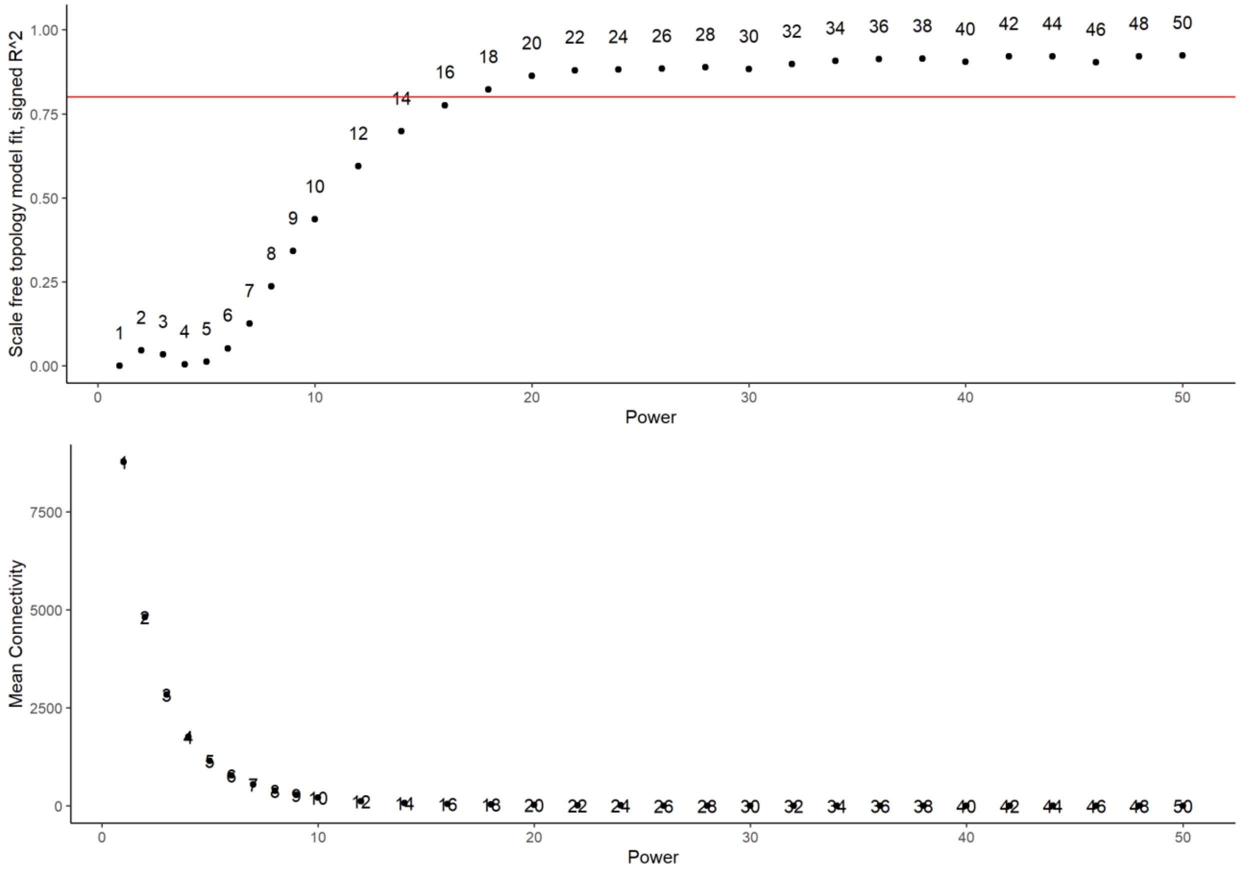

Supplementary Figure 1. Soft threshold power of signed weighted gene correlation network analysis model of co-expressed genes in MCC transcriptomes (soft threshold 18 chosen as being above signed R^2 threshold above 0.8 while maximizing mean connectivity).

Supplementary Figure 2

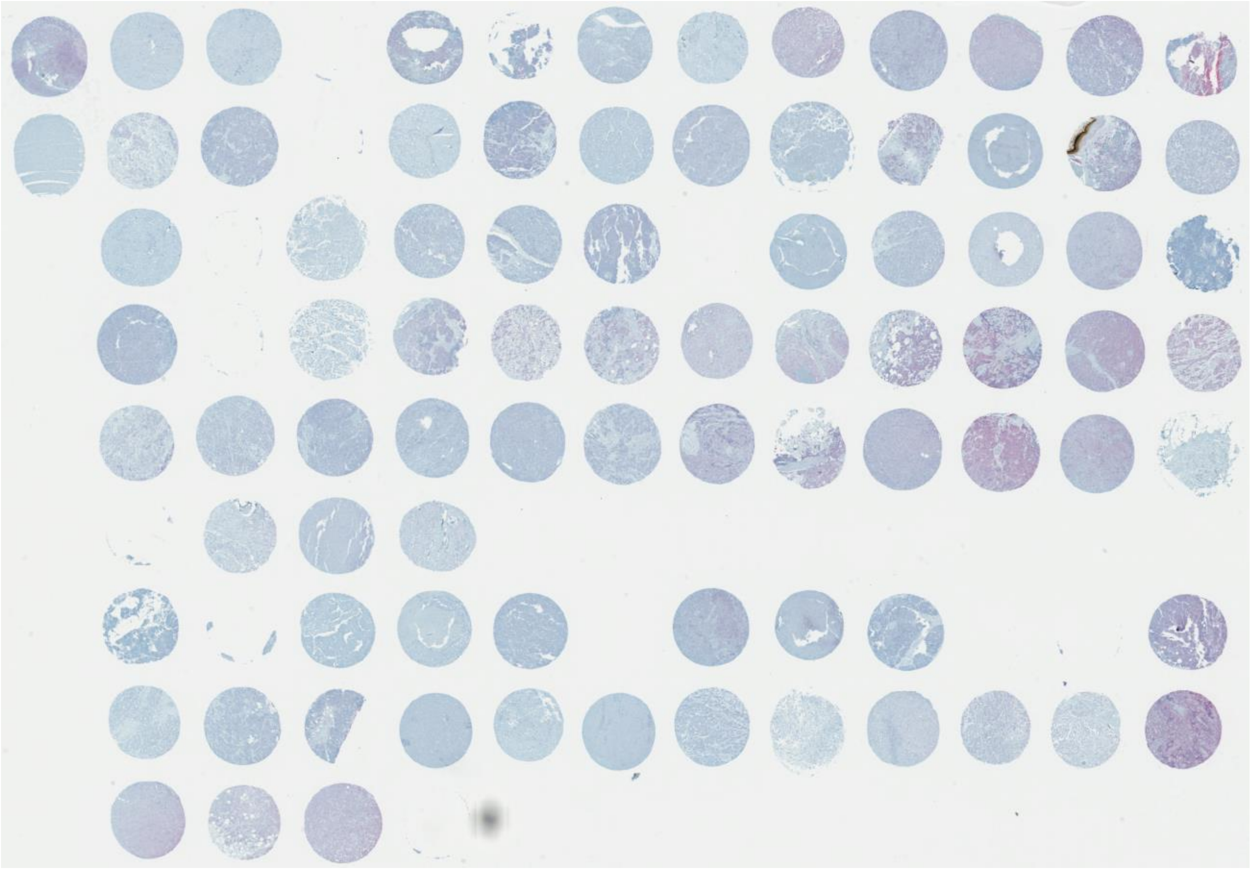

Supplementary Figure 2. Tissue microarray of BCMA protein expression in MCC FFPE samples and two control samples (top left: tonsil [top], colon [bottom]).

Supplementary Figure3

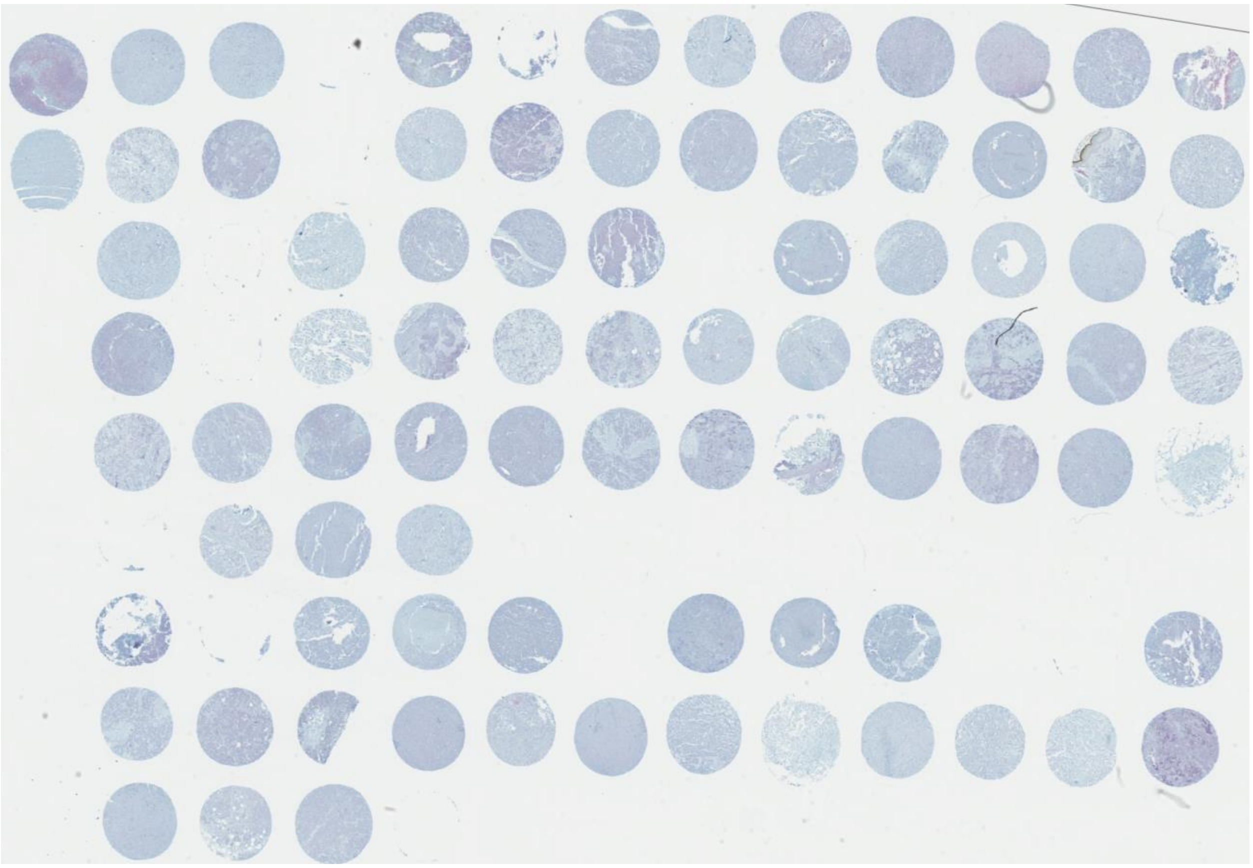

Supplementary Figure 3. Tissue microarray of CD10 protein expression in MCC FFPE samples and two control samples (top left: tonsil [top], colon [bottom]).

Supplementary Figure 4

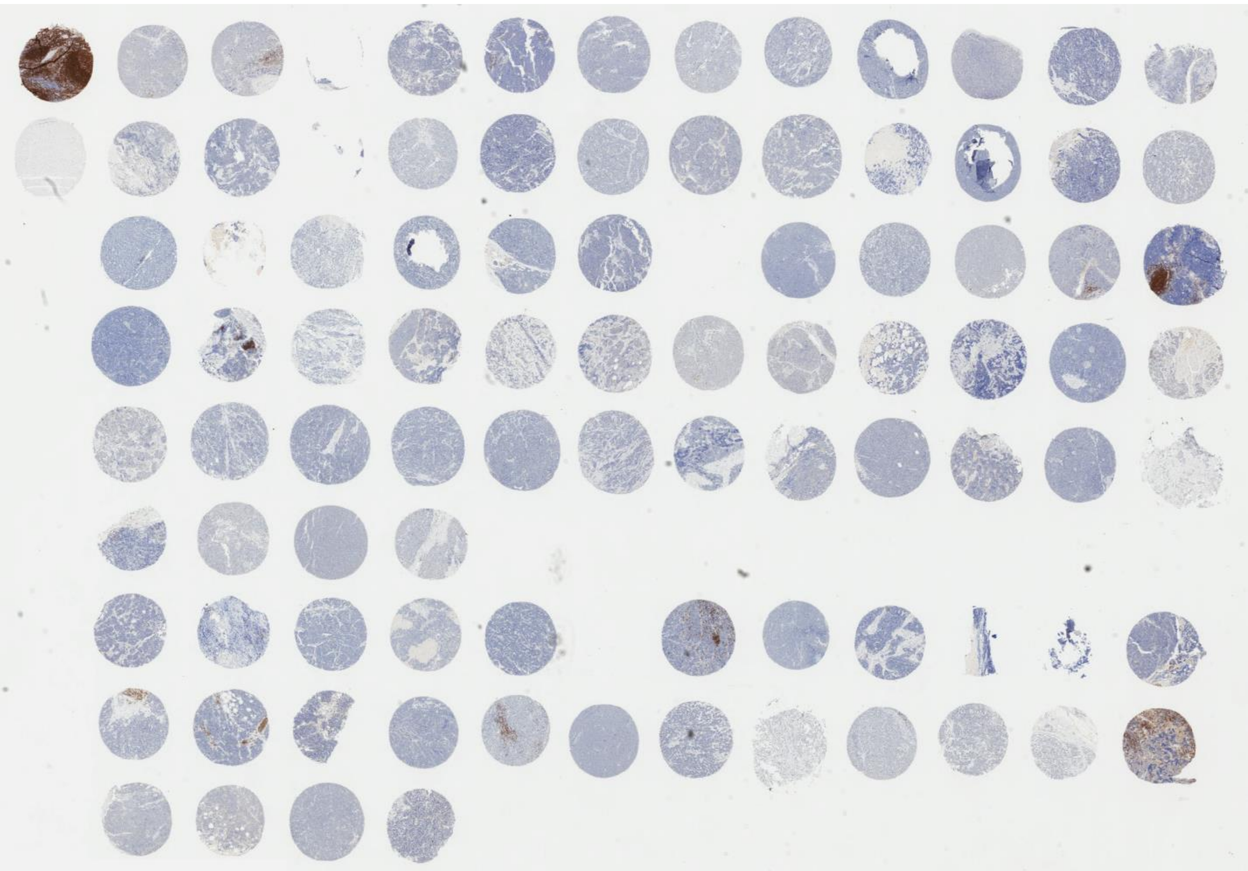

Supplementary Figure 4. Tissue microarray of CD19 protein expression in MCC FFPE samples and two control samples (top left: tonsil [top], colon [bottom]).

Supplementary Figure 5

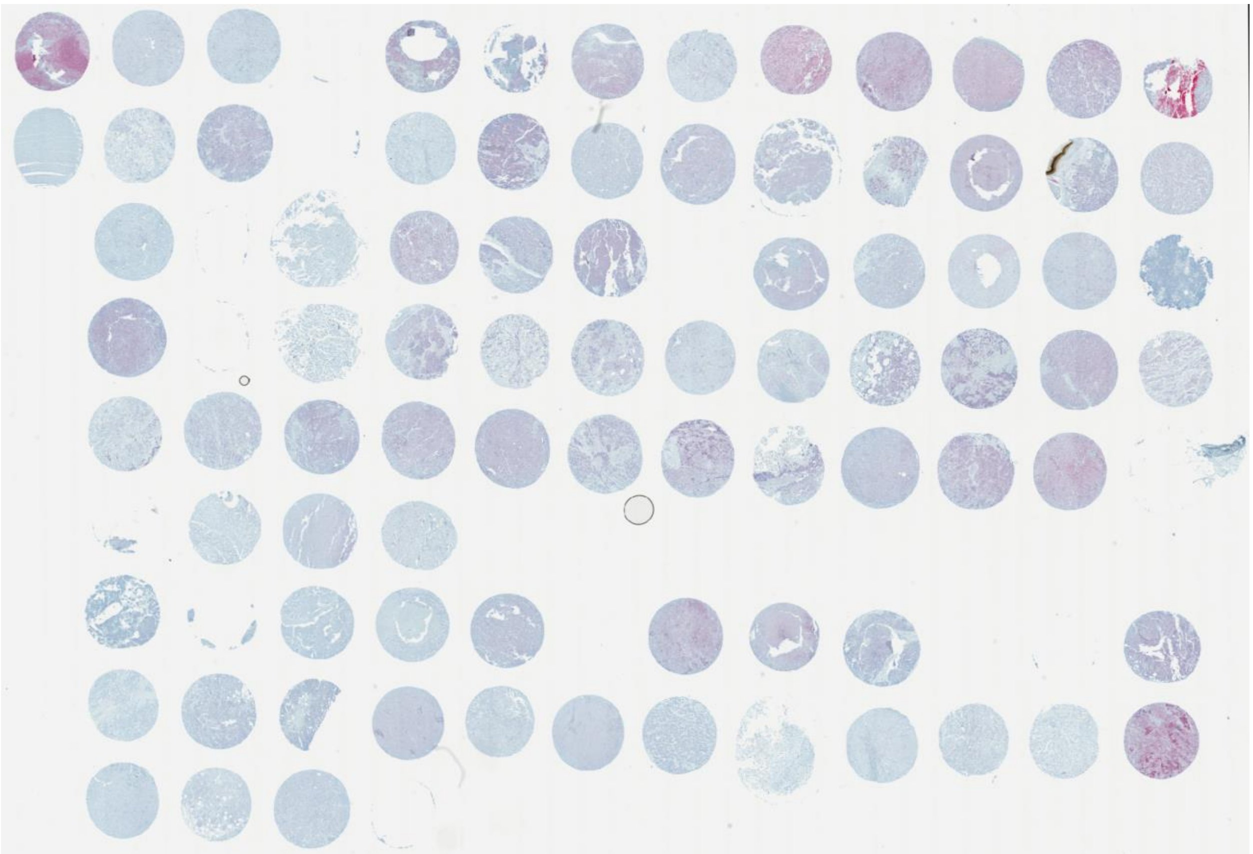

Supplementary Figure 5. Tissue microarray of CD93 protein expression in MCC FFPE samples and two control samples (top left: tonsil [top], colon [bottom]).

Supplementary Figure 6

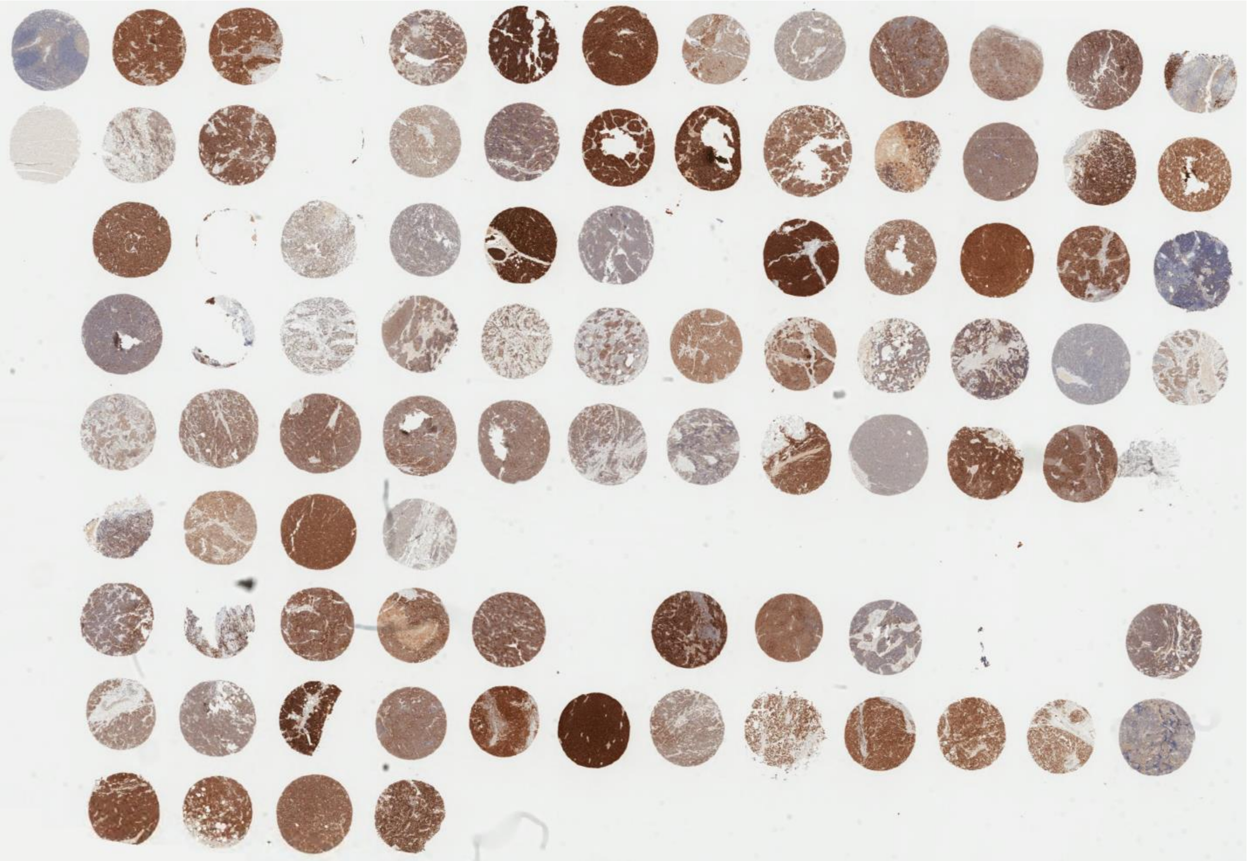

Supplementary Figure 6. Tissue microarray of Chromogranin A protein expression in MCC FFPE samples and two control samples (top left: tonsil [top], colon [bottom]).

Supplementary Figure 7

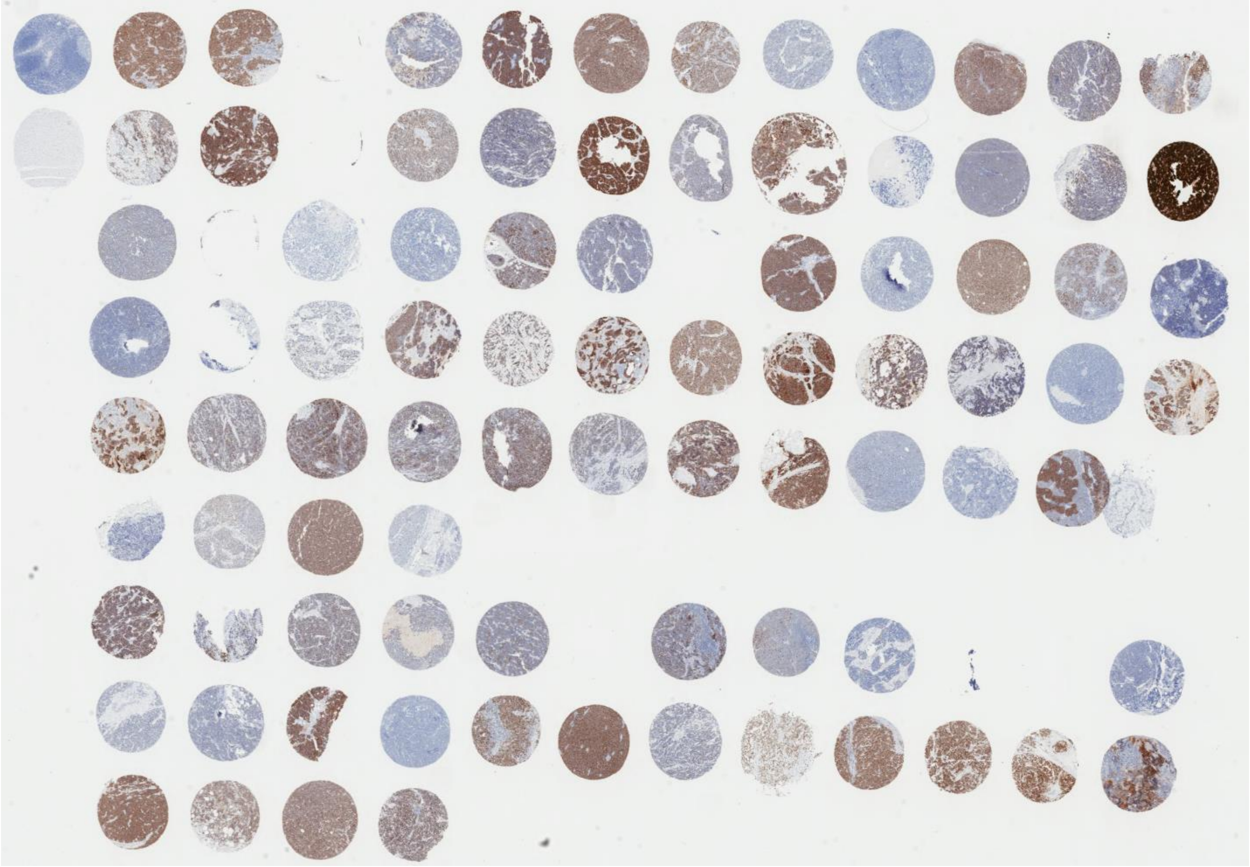

Supplementary Figure 7. Tissue microarray of CK20 protein expression in MCC FFPE samples and two control samples (top left: tonsil [top], colon [bottom]).

Supplementary Figure 8

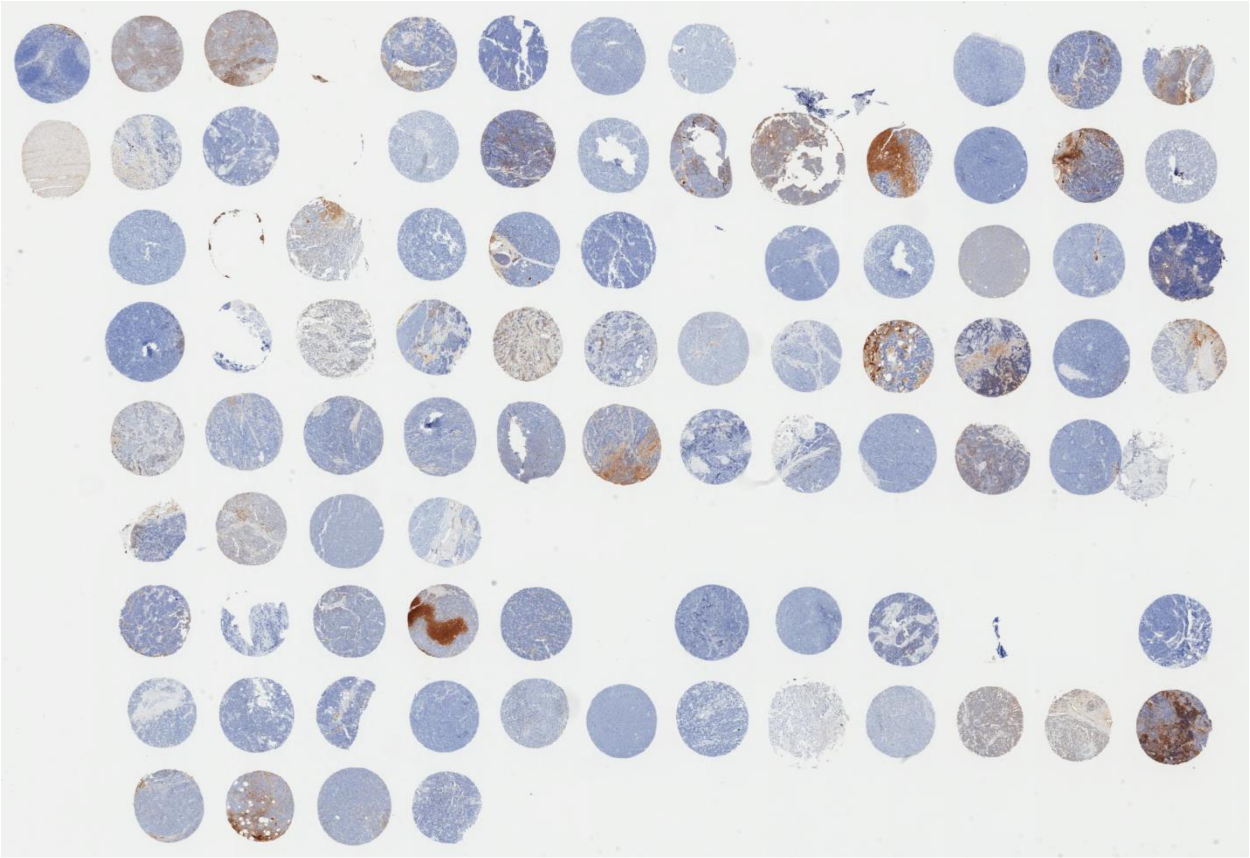

Supplementary Figure 8. Tissue microarray of IgA protein expression in MCC FFPE samples and two control samples (top left: tonsil [top], colon [bottom]).

Supplementary Figure 9

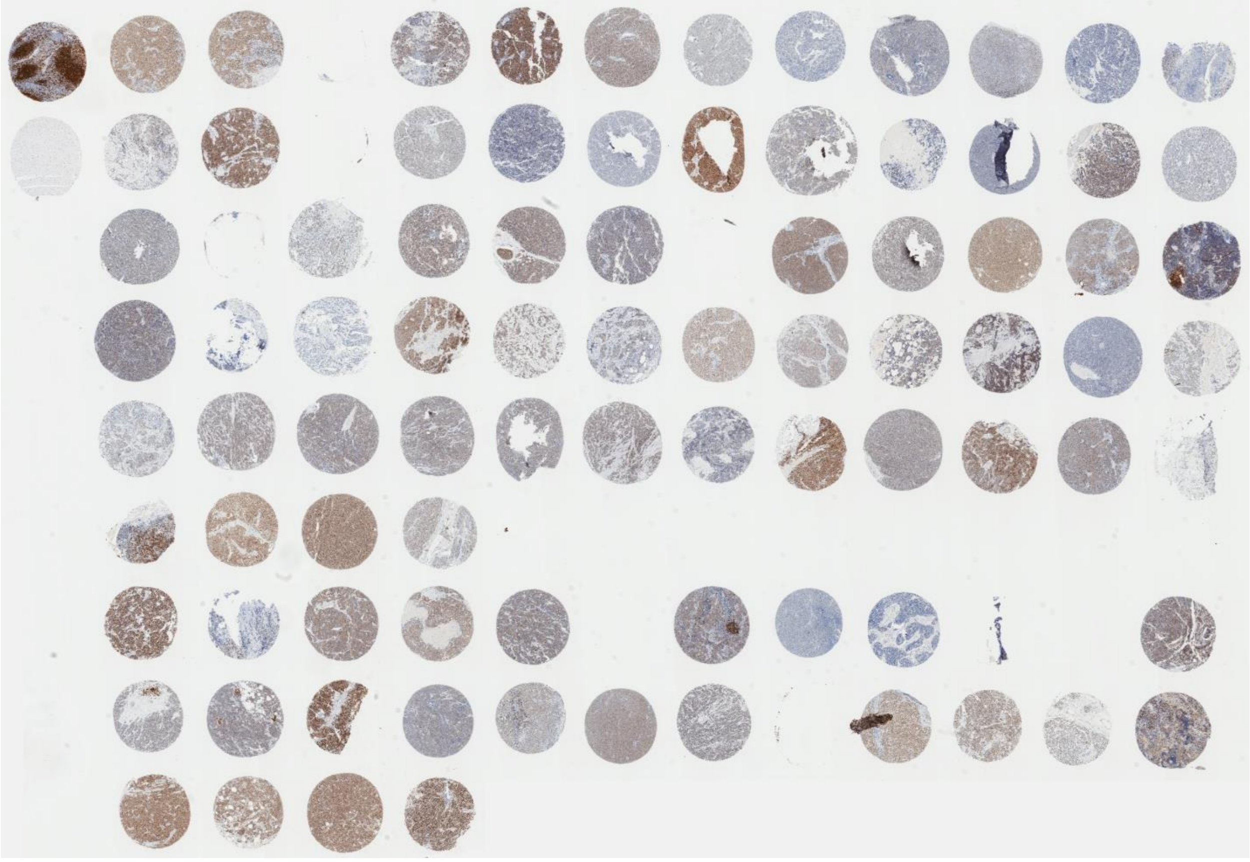

Supplementary Figure 9. Tissue microarray of PAX5 protein expression in MCC FFPE samples and two control samples (top left: tonsil [top], colon [bottom]).

Supplementary Figure 10

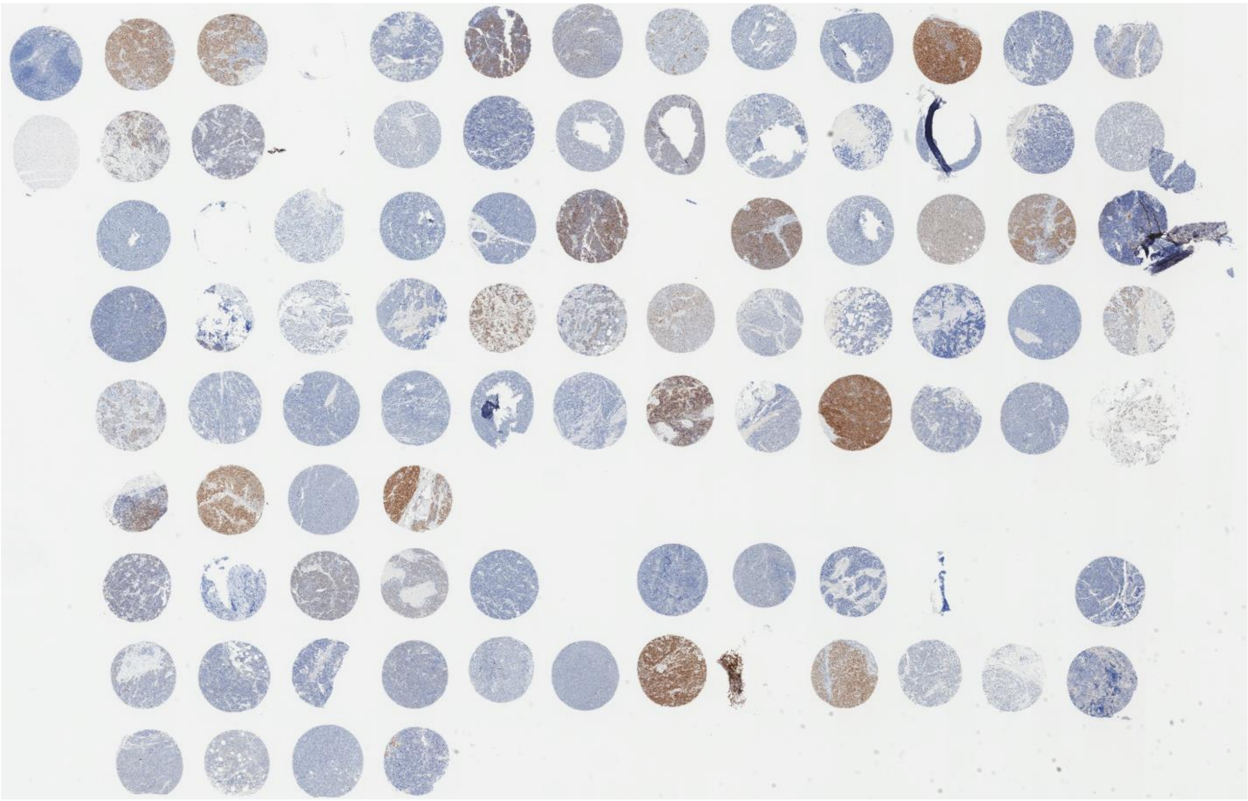

Supplementary Figure 10. Tissue microarray of TdT protein expression in MCC FFPE samples and two control samples (top left: tonsil [top], colon [bottom]).

Supplementary Figure 11

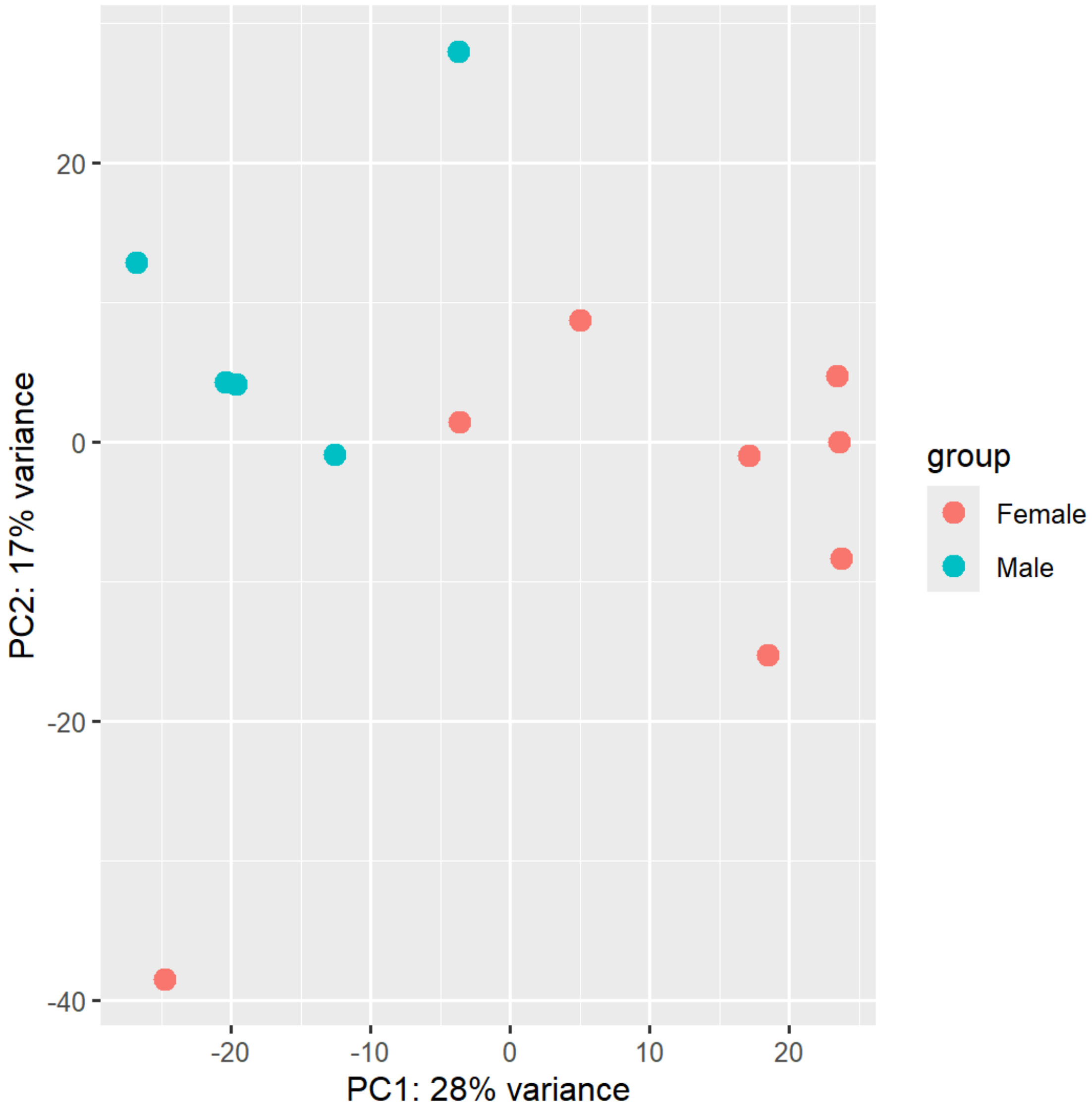

Supplementary Figure 11. Principal component analysis of MCC transcriptomes based on patient sex.

Supplementary Figure 12

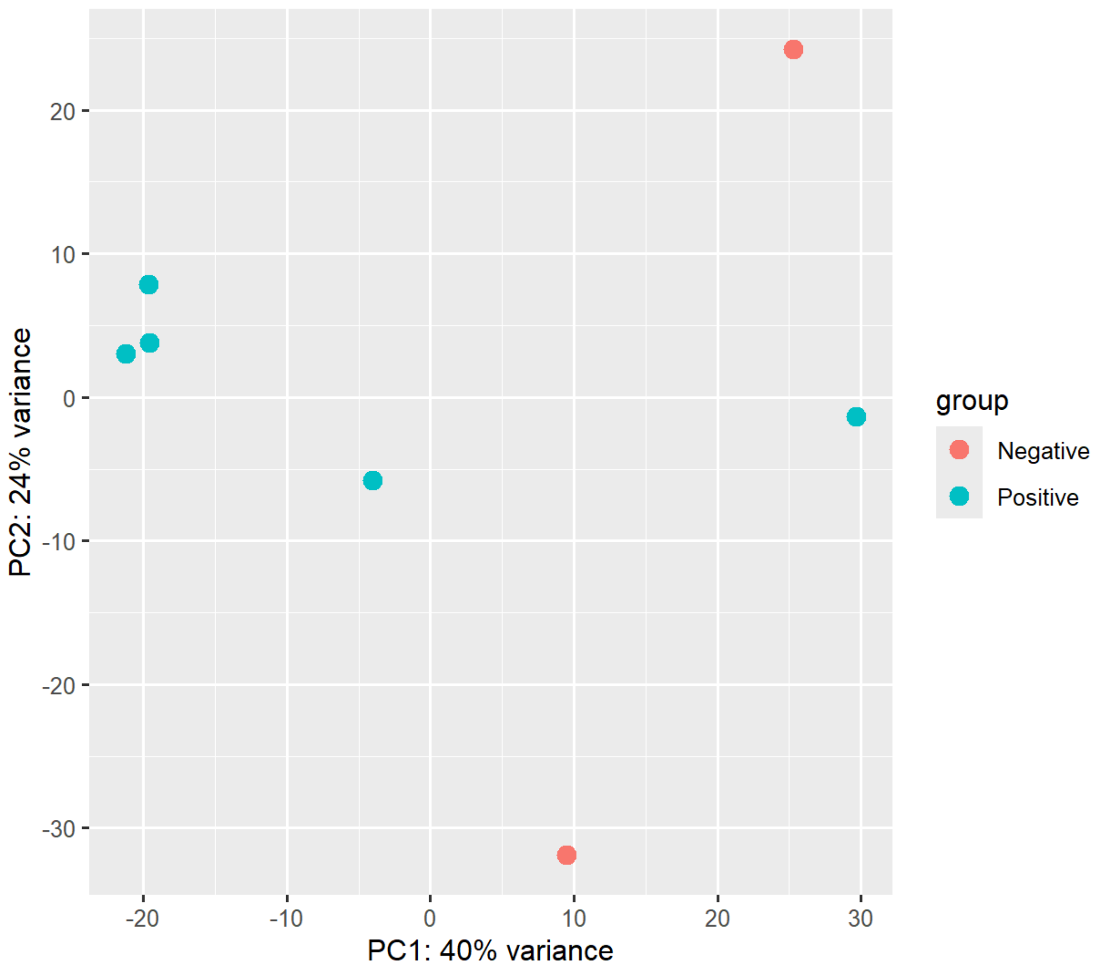

Supplementary Figure 12. Principal component analysis of MCC transcriptomes based on patient Merkel cell polyoma virus status.

Supplementary Figure 13

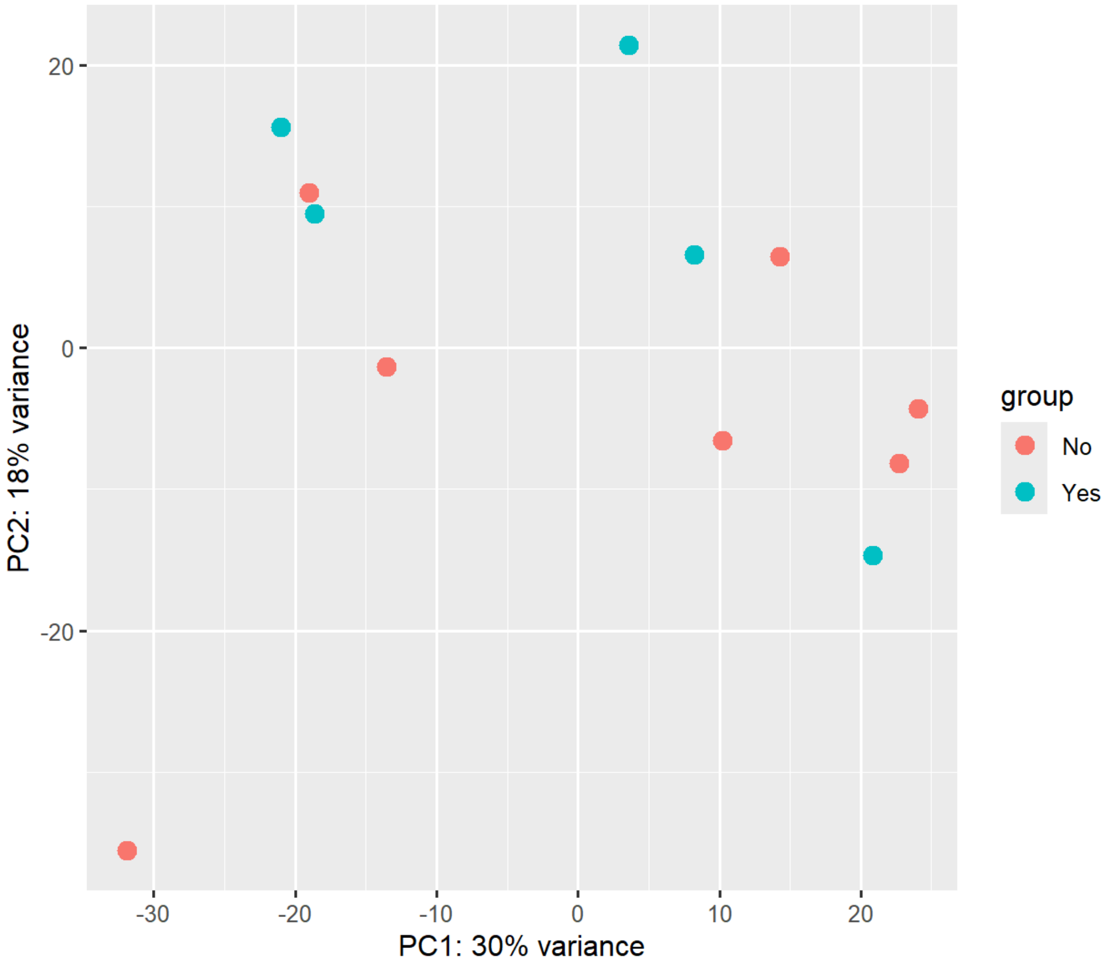

Supplementary Figure 13. Principal component analysis of MCC transcriptomes based on patient immunosuppressed status.

Supplementary Figure 14

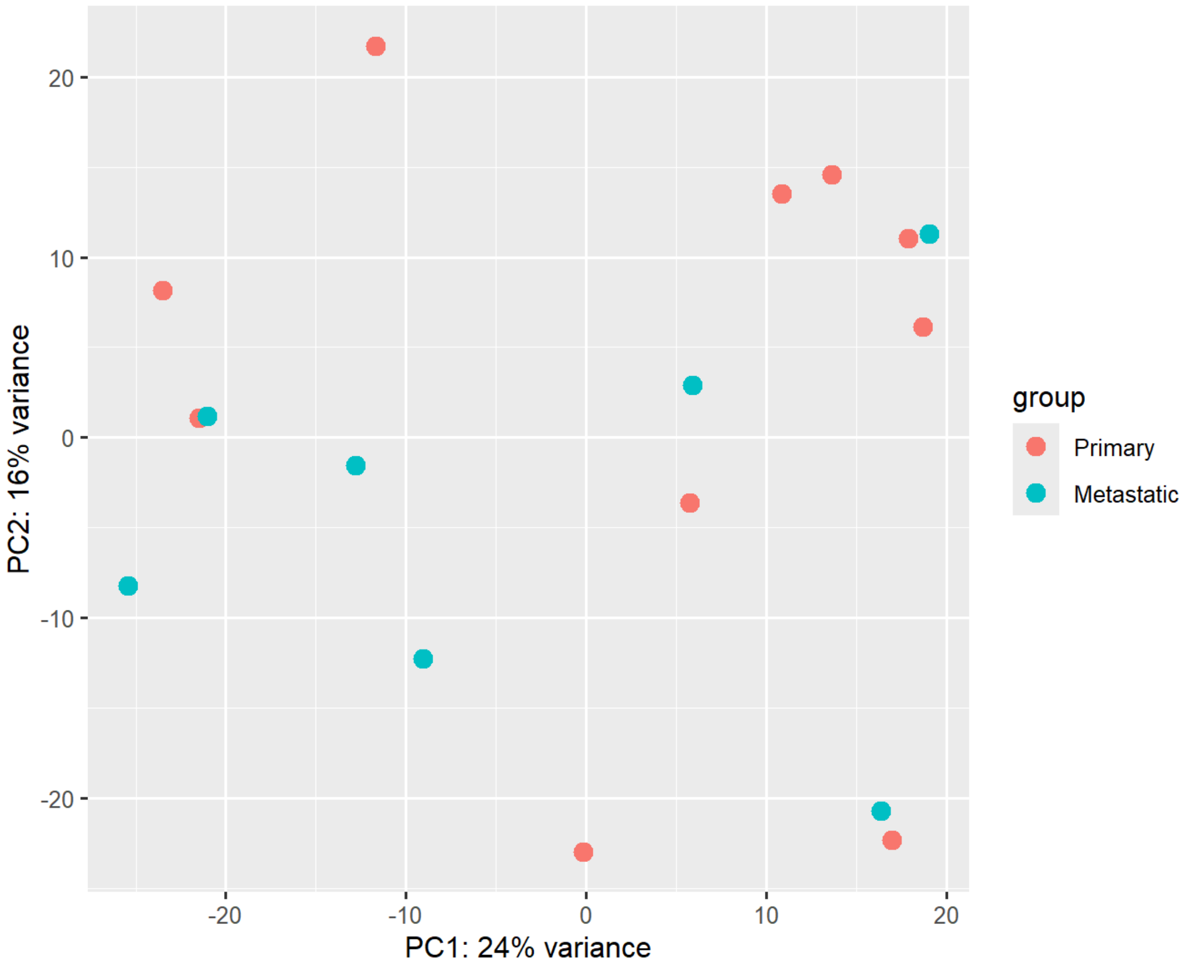

Supplementary Figure 14. Principal component analysis of MCC transcriptomes based on patient tumor type.

Supplementary Figure 15

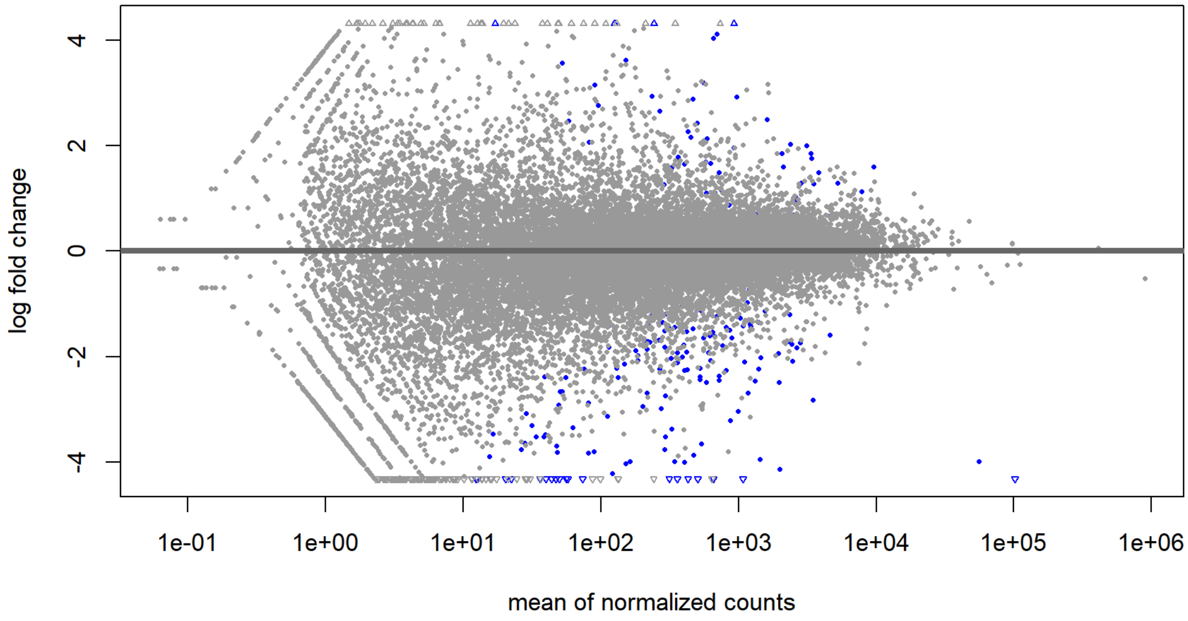

Supplementary Figure 15. Minus-average plot of differential gene expression of MCC transcriptomes based on patient sex. Blue markers indicate significant differentially expressed genes.

Supplementary Figure 16

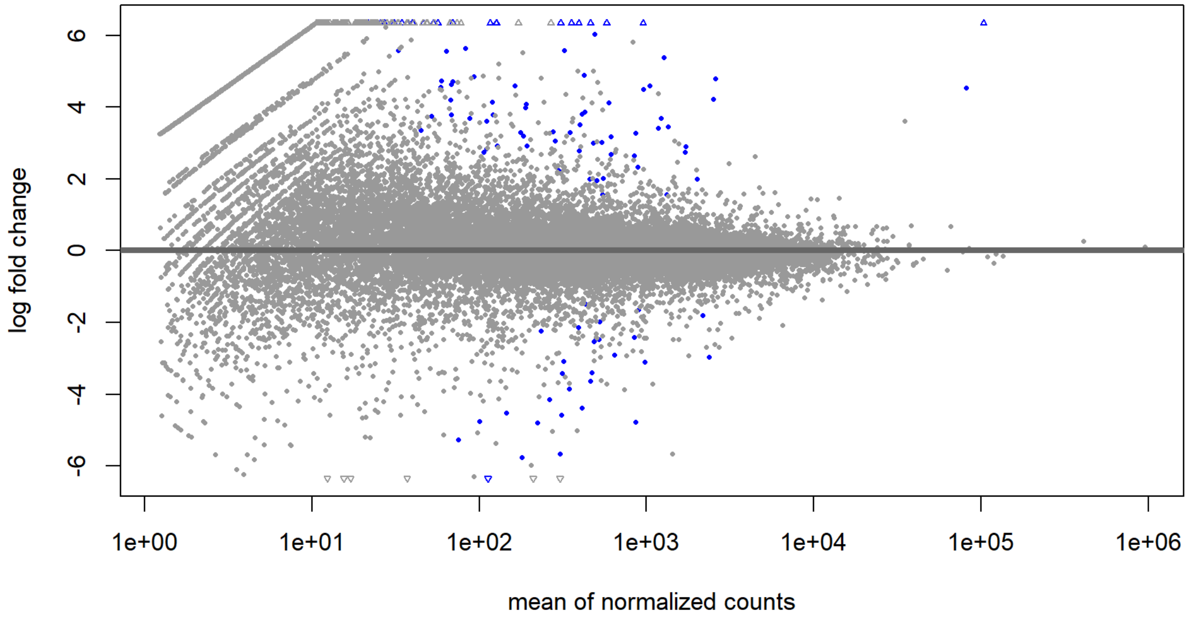

Supplementary Figure 16. Minus-average plot of differential gene expression of MCC transcriptomes based on patient Merkel cell polyoma virus status. Blue markers indicate significant differentially expressed genes.

Supplementary Figure 17

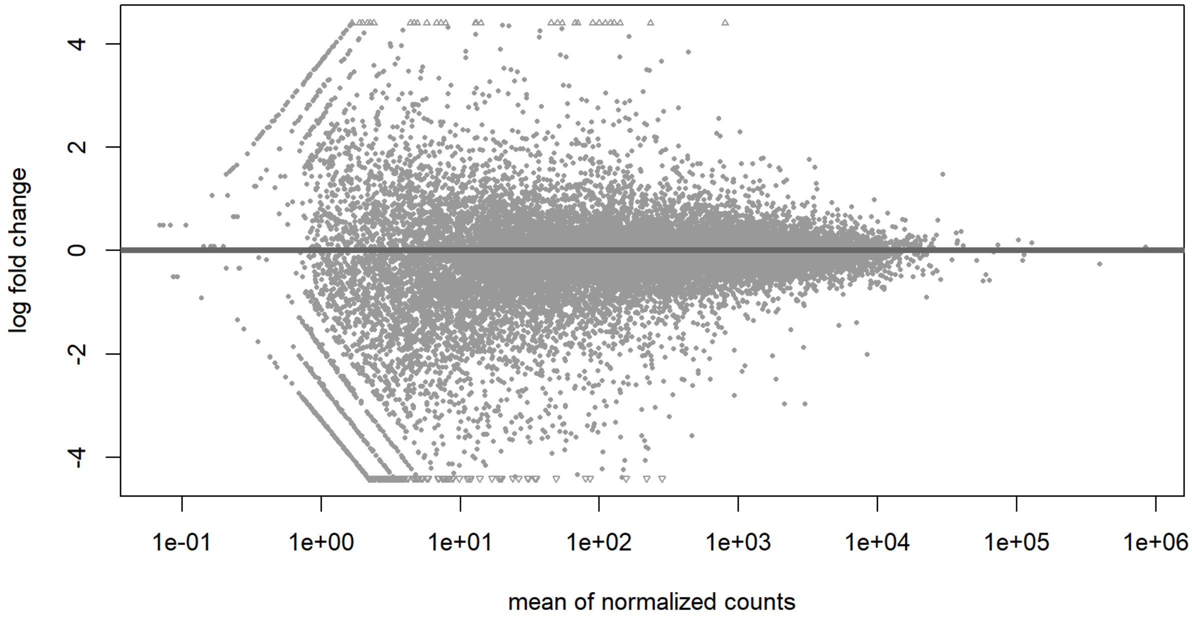

Supplementary Figure 17. Minus-average plot of differential gene expression of MCC transcriptomes based on patient immunosuppressed status.

Supplementary Figure 18

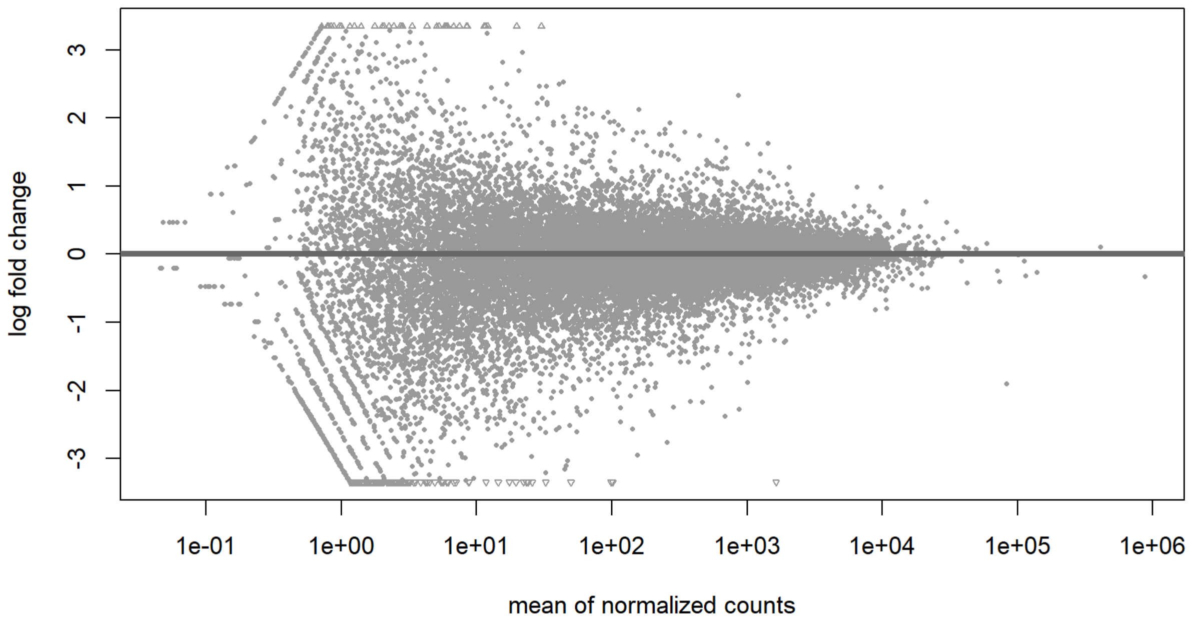

Supplementary Figure 18. Minus-average plot of differential gene expression of MCC transcriptomes based on patient tumor type.

Supplementary Figure 19

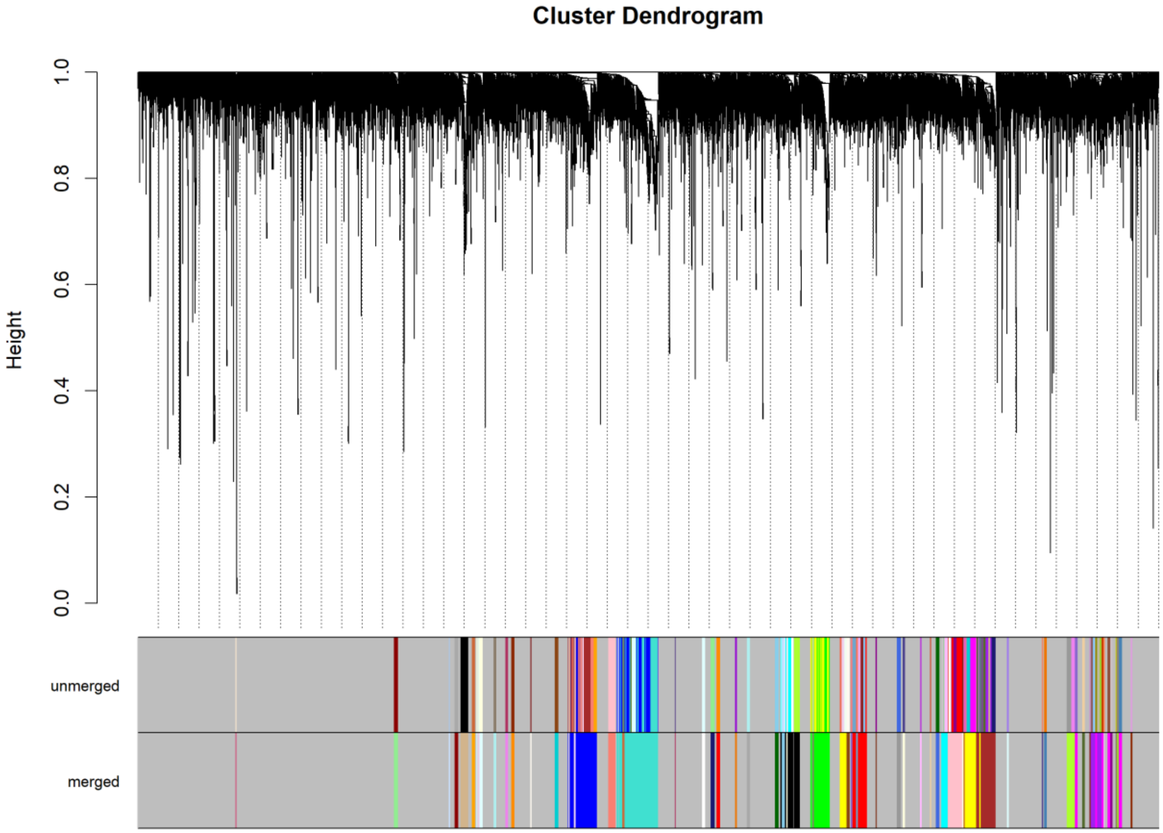

Supplementary Figure 19. Cluster dendrogram of 57 co-expression modules across 17,531 genes based on weighted gene correlation network model of MCC transcriptomes.

Supplementary Figure 20

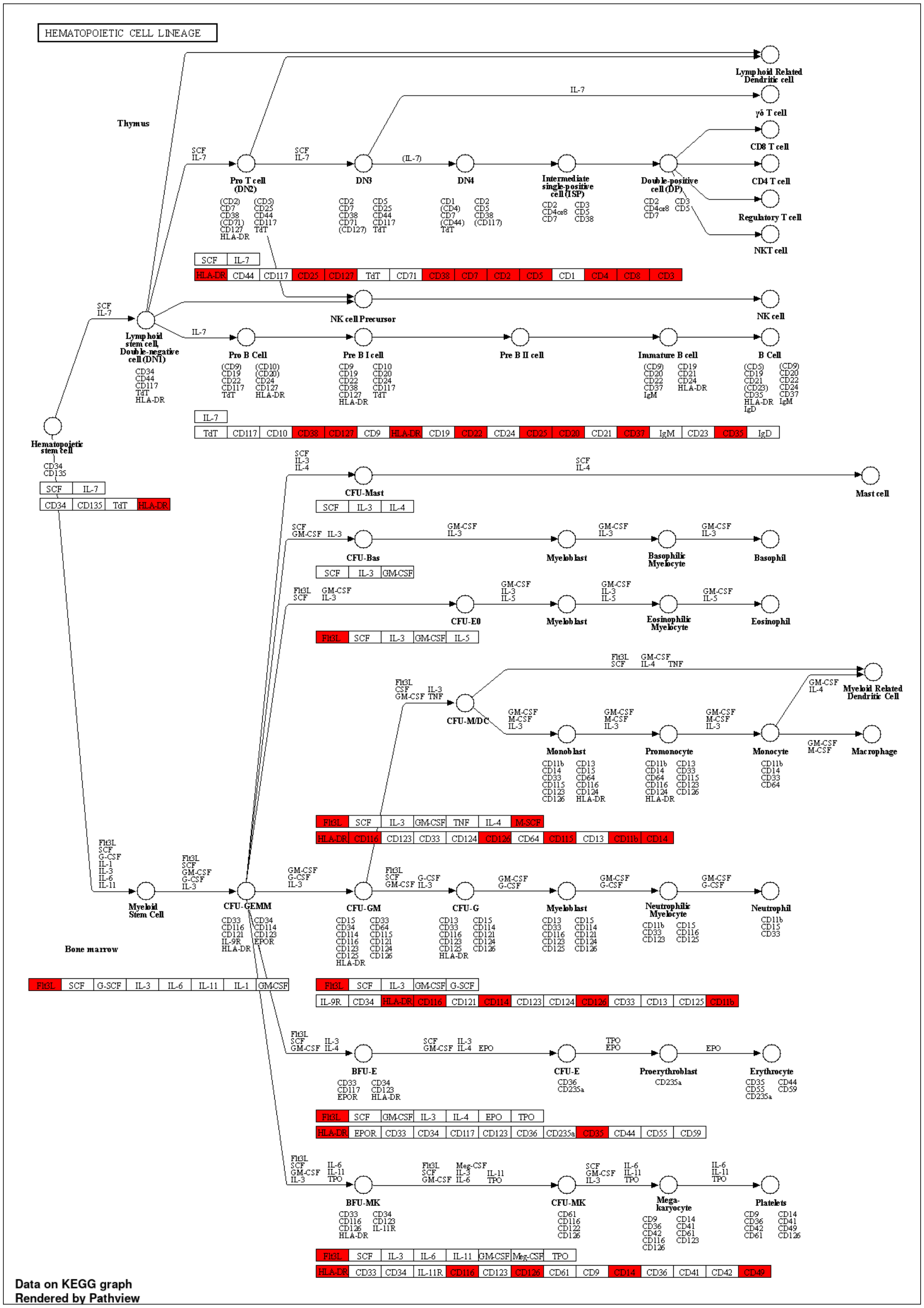

Supplementary Figure 20. KEGG enrichment analysis of MCC-expressed gene correlation network (turquoise module) shows expressed genes in hematopoietic stem cell lineage pathway (hsa04640).

Supplementary Figure 21

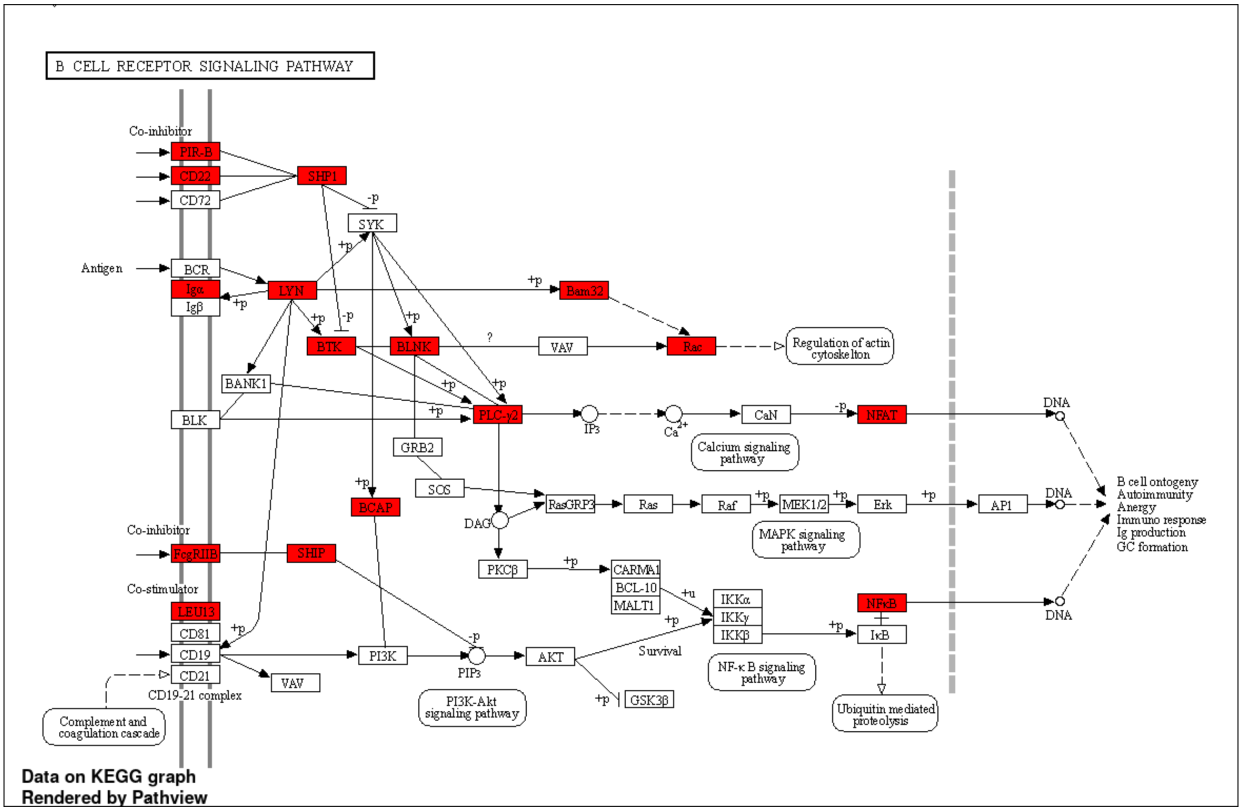

Supplementary Figure 21. KEGG enrichment analysis of MCC-expressed gene correlation network (turquoise module) shows expressed genes in B-cell receptor signaling pathway (hsa04662).

Supplementary Figure 22

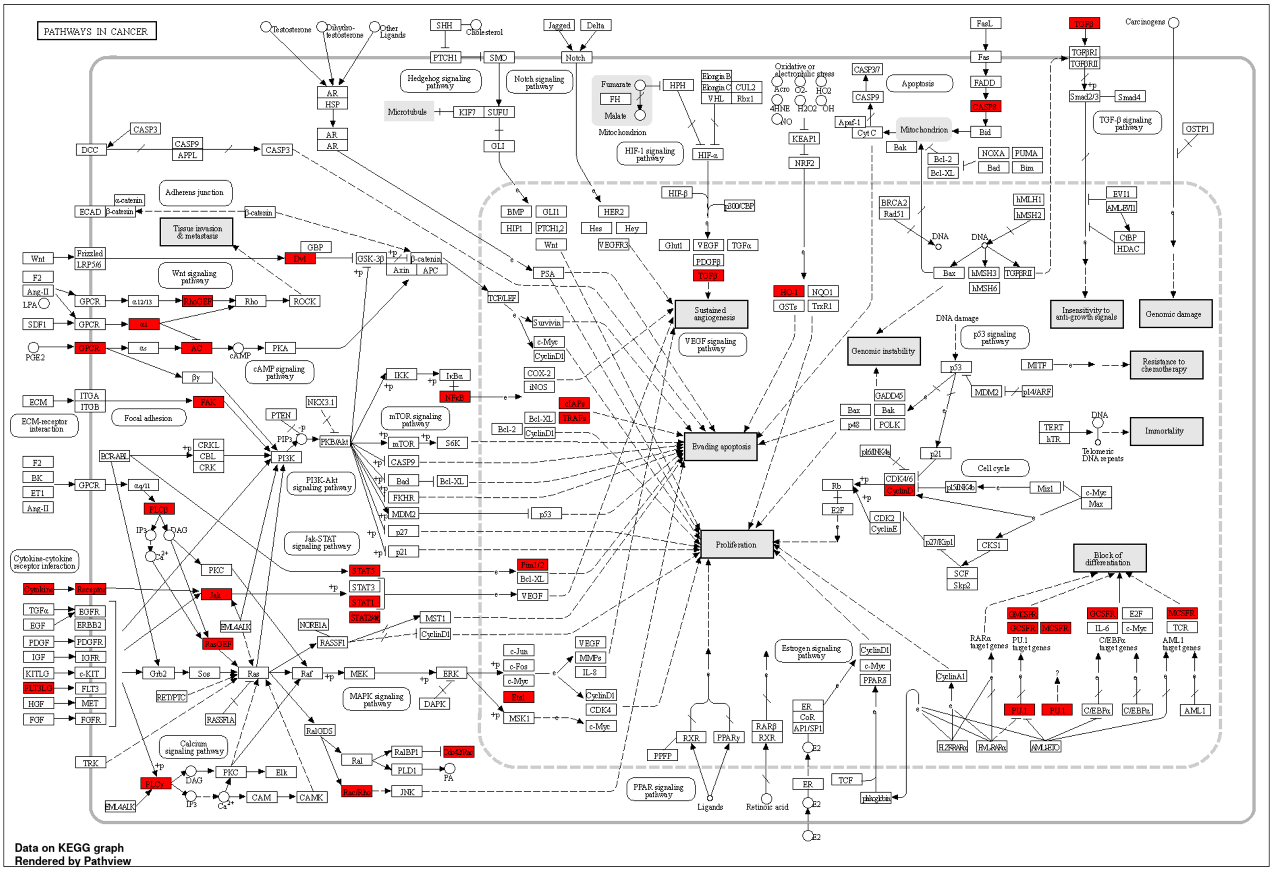

Supplementary Figure 22. KEGG enrichment analysis of MCC-expressed gene correlation network (turquoise module) shows expressed genes in pathways in cancer pathway (hsa05200).
