# Supplementary material for: Investigating the cell of origin and novel molecular targets in Merkel cell carcinoma: a historic misnomer
Source: Mol Oncol. 2025 Aug 5;20(2):331–47. doi: 10.1002/1878-0261.70107 (PMC12936429; doi:10.1002/1878-0261.70107)
Supplement: Supplementary file 2 — Table S1. Overview of Merkel cell carcinoma patient demographics. [file MOL2-20-331-s002.docx]

**Supplementary Table 1.** Overview of Merkel cell carcinoma patient demographics. NR- not reported.

| Patient ID | Age | Sex | Race | Immunosuppression (n) | Primary Location | Merkel Cell Polyoma Virus Status | Presence of Metastases (Y/N) |
| --- | --- | --- | --- | --- | --- | --- | --- |
| *MCC34* | 82 | F | Caucasian | N | Right arm | Negative | N |
| *MCC33* | 89 | M | Caucasian | N | Left elbow | NR | N |
| *MCC28* | 81 | F | Caucasian | N | Right ear | NR | N |
| *MCC40* | 88 | M | Caucasian | N | Right forearm | NR | N |
| *MCC48* | 80 | M | Caucasian | N | Right arm | Positive | N |
| *MCC41* | 77 | M | Caucasian | Y: transplant (1) | Left lower back | Positive | N |
| *MCC42* | 72 | M | NR | NR | Elbow | NR | N |
| *MCC43* | 82 | F | Caucasian | N | Right wrist | Positive | N |
| *MCC44* | 92 | M | Caucasian | N | Lower lip | NR | N |
| *MCC45* | 66 | F | Caucasian | N | Right dorsal hand | Positive | N |
| *MCC46* | 86 | M | Caucasian | Y: IBD (1) | Scalp | Positive | N |
| *MCC47* | 84 | F | Caucasian | N | Left cheek | Negative | N |
| *MCC36* | 90 | M | Caucasian | NR | Scalp | NR | N |
| *MCC37* | 90 | M | Caucasian | NR | Right temple | NR | N |
| *MCC38* | 52 | F | Caucasian | N | Right buttock | Negative | N |
| *MCC61* | 78 | M | Caucasian | N | Right thigh | Positive | N |
| *MCC57* | 73 | F | Caucasian | Y: arthritis (1) | Right arm | NR | N |
| *MCC56* | 77 | M | Caucasian | Y: transplant (1) | Left Buttock | Positive | N |
| *MCC55* | 85 | M | Caucasian | N | Left temple | Negative | N |
| *MCC58* | 72 | M | Caucasian | N | Right anterolateral arm | Positive | N |
| *MCC64* | 84 | F | Caucasian | Y | Left upper arm | Positive | N |
| *MCC50* | 86 | M | Caucasian | Y: IBD (1); melanoma (1) | Scalp | Negative | N |
| *MCC63* | NR | NR | NR | NR | Abdomen/flank | NR | N |
| *MCC60* | 79 | M | NR | NR | Right lateral thigh | Positive | N |
| *MCC62* | 78 | M | Caucasian | N | Right thigh, biopsy | Positive | N |
| *MCC59* | 92 | M | Asian | N | Left upper cheek | Negative | N |
| *MCC32* | 76 | F | NR | NR | Arm | NR | N |
| *MCC29* | 82 | M | Caucasian | Y | Left temple | NR | N |
| *MCC35* | 90 | F | Caucasian | N | Left foot | Positive | N |
| *MCC31* | 81 | F | Caucasian | N | Left neck | Positive | N |
| *MCC30* | 81 | M | Caucasian | N | Left calf | Positive | N |
| *MCC39* | 52 | F | Caucasian | N | Negative | NR | N |
| *MCC24* | 79 | M | Caucasian | N | Periorbital | Positive | N |
| *MCC16* | 67 | M | Caucasian | Y: transplant (1) | Left arm | Positive | N |
| *MCC17* | 84 | F | Caucasian | N | Right ankle | NR | N |
| *MCC18* | 86 | M | Caucasian | NR | Left ear | NR | N |
| *MCC23* | 82 | M | Caucasian | Y | Left cheek | NR | N |
| *MCC26* | 91 | M | Caucasian | N | Finger | NR | N |
| *MCC19* | NR | F | Caucasian | NR | Thigh | NR | N |
| *MCC21* | 68 | F | Caucasian | N | Right elbow | NR | N |
| *MCC27* | 68 | F | Caucasian | N | Right elbow | NR | N |
| *MCC20* | 72 | M | Caucasian | N | Right posterior arm | NR | N |
| *MCC25* | 82 | M | Caucasian | Y | Left Cheek | NR | N |
| *MCC22* | 81 | M | Caucasian | N | Right arm | Positive | N |
| *MCC5* | 66 | F | Asian | N | Left tonsil | NR | N |
| *MCC12* | 38 | M | NR | NR | Stomach wall | NR | N |
| *MCC1* | NR | M | NR | NR | Left forearm | NR | N |
| *MCC3* | 66 | M | Caucasian | Y: transplant (1) | Left upper shoulder | NR | N |
| *MCC6* | NR | M | NR | NR | Upper lip | NR | N |
| *MCC11* | NR | M | NR | NR | Right shoulder | NR | N |
| *MCC10* | 85 | M | Caucasian | N | Right cheek | NR | N |
| *MCC4* | NR | M | NR | NR | Right knee | NR | N |
| *MCC13* | 66 | F | NR | NR | Left forearm | NR | N |
| *MCC14* | NR | M | NR | NR | Left Buttock | NR | N |
| *MCC15* | NR | F | NR | NR | Left thigh | NR | N |
| *MCC8* | NR | M | NR | NR | Left cheek | NR | N |
| *MCC7* | NR | F | NR | NR | Arm | NR | N |
| *MCC74* | 69 | F | NR | NR | Left axilla | NR | N |
| *MCC65* | 74 | M | Caucasian | N | Mouth | NR | Y |
| *MCC66* | 66 | F | Asian | N | Right medial elbow | NR | Y |
| *MCC67* | NR | NR | NR | NR | Left inguinal lymph node | NR | Y |
| *MCC68* | 66 | M | Caucasian | N | Left iliac, obturator lymph node | NR | Y |
| *MCC69* | 60 | F | NR | NR | Left axillary lymph node | NR | Y |
| *MCC70* | 79 | M | Caucasian | N | Liver | Positive | Y |
| *MCC71* | 74 | M | Caucasian | N | Left sentinel lymph node | NR | Y |
| *MCC72* | 76 | M | Caucasian | N | Left thigh, groin | NR | Y |
| *MCC73* | 50 | F | Middle east | N | Right neck | NR | Y |
| *MCC75* | 73 | M | Caucasian | Y: cancer (1) | Right axilla, elbow | NR | Y |
| *MCC76* | 89 | M | Caucasian | Y: malignancy (1) | Left axillary lymph node | NR | Y |
| *MCC77* | 73 | M | Caucasian | Y: cancer (1) | Wrist | NR | Y |
| *MCC79* | 82 | F | Caucasian | N | Left neck | Negative | Y |
| *MCC80* | 54 | F | Caucasian | N | Right neck | NR | Y |
| *MCC81* | 80 | M | Caucasian | N | Right calf | NR | Y |
| *MCC84* | 75 | F | Caucasian | N | Right groin, right leg | Positive | Y |
| *MCC86* | 77 | M | Caucasian | Y: transplant (1) | Left groin, left back | Positive | Y |
| *MCC88* | 75 | F | Caucasian | Y: IBD (1) | Right neck | Positive | Y |
| *MCC89* | 77 | M | Caucasian | Y: transplant (1) | Left inguinal skin | Positive | Y |
| *MCC90* | 77 | M | Caucasian | Y: transplant (1) | Left flank, left lumbar, left parasternal | Positive | Y |
| *MCC91* | 77 | M | Caucasian | Y: transplant (1) | Left flank | Positive | Y |
| *MCC92* | 82 | F | Caucasian | N | Right lower leg | NR | Y |
| *MCC83* | NR | NR | NR | NR | NR | NR | Y |
| *MCC100* | NR | NR | NR | NR | NR | NR | NR |
| *MCC101* | NR | NR | NR | NR | NR | NR | NR |
| *MCC102* | NR | NR | NR | NR | NR | NR | NR |
| *MCC103* | NR | NR | NR | NR | NR | NR | NR |
| *MCC104* | NR | NR | NR | NR | NR | NR | NR |
| *MCC105* | NR | NR | NR | NR | NR | NR | NR |
| *MCC106* | NR | NR | NR | NR | NR | NR | NR |
| *MCC107* | NR | NR | NR | NR | NR | NR | NR |
| *MCC108* | NR | NR | NR | NR | NR | NR | NR |
| *MCC109* | NR | NR | NR | NR | NR | NR | NR |
| *MCC110* | NR | NR | NR | NR | NR | NR | NR |
