# Supplementary material for: Investigating the cell of origin and novel molecular targets in Merkel cell carcinoma: a historic misnomer
Source: Mol Oncol. 2025 Aug 5;20(2):331–47. doi: 10.1002/1878-0261.70107 (PMC12936429; doi:10.1002/1878-0261.70107)
Supplement: Supplementary file 4 — Table S3. Summary of potential novel therapeutic targets for Merkel cell carcinoma. [file MOL2-20-331-s005.docx]

**Supplementary Table 3.** Summary of potential novel therapeutic targets for Merkel cell carcinoma.

| Target | Expression observed in MCC | Biologic role | Existing/emerging therapies |
| --- | --- | --- | --- |
| CD19 | Expression observed by western blotting, immunohistochemistry (in 19.6% of cases) and RNA-Sequencing | CD19 is a transmembrane receptor often expressed in Pro-B, pre B cells, mature, and activated B cells where it functions in B-cell development as well as B-cell activation by working with the B cell receptor to lower threshold for activation. | Multiple US FDA therapies have been approved targeting CD19 including (CAR) T-cell treatments: Tisagenlecleucel (Kymriah), approved for relapsed or refractory B-cell acute lymphoblastic leukemia (ALL) and certain types of non-Hodgkin lymphoma, Axicabtagene ciloleucel (Yescarta), approved for relapsed or refractory large B-cell lymphoma, Obecabtagene autoleucel (Aucatzyl), recently approved for relapsed or refractory B-cell precursor ALL; bispecific T-cell engagers (BiTEs): Blinatumomab (Blincyto), approved for the treatment of relapsed or refractory B-cell precursor ALL and has been shown to induce remission in  a patients with otherwise untreatable B-cell cancers; and antibody-drug conjugates (ADCs): Loncastuximab tesirine (Zynlonta), approved for relapsed or refractory large B-cell lymphoma and high-grade B-cell lymphoma[1-3] |
| BCMA (B-cell maturation antigen, TNFRSF17) | Expression observed by immunohistochemistry (in 62% of cases) and RNA-Sequencing | BCMA plays a critical role in the development of B cells to plasma cells. It acts as a transmembrane protein that primarily functions as a survival signal for plasma cells. It is also highly expressed in Multiple Myeloma. | Multiple FDA approved therapies including BCMA-targeting CAR-T therapies (Idecabtagene vicleucel, Ciltacabtagene autoleucel) approved for the treatment of relapsed or refractory treatment of Multiple Myeloma after ≥4 prior therapies and bispecific antibodies such as Teclistamab (approved for the treatment of relapsed or refractory treatment of Multiple Myeloma after ≥4 prior therapies) and Elranatamab under ongoing clinical trial investigation for Multiple Myeloma.[1, 4, 5] |
| CD93 | Expression observed by immunohistochemistry (in 57.6% of cases) and RNA-Sequencing | CD93 is a transmembrane glycoprotein involved in a variety of biological processes, including cell adhesion (of leukocytes to endothelial cells), inflammation, angiogenesis, and immune regulation. It is expressed on several cell types, including myeloid cells (monocytes, macrophages), endothelial cells, and certain progenitor cells. In innate immunity it plays a role in regulating phagocytosis. | Under investigation as a therapeutic target in AML but no approved drugs.  Potential therapies include: NOT-gated CD93 CAR-T treatment for AML [6]; CD93 monoclonal antibodies (mAbs) targeting for AML and solid tumors respectively [7, 8]. |
| TdT (Terminal deoxynucleotidyl transferase) | Expression observed by western blotting, immunohistochemistry (in 33.7% of cases) and RNA-Sequencing | TdT is a DNA polymerase enzyme which is involved in V(D)J recombination of immunoglobulin and T-cell receptor genes to create diversity within the immune system. | There are no approved direct therapies for TdT but it has been proposed as a promising therapeutic target in B-ALL via T cell receptor (TCR)-modified T-cells targeting TdT [9]. |

References:

1. Goyco Vera, D., et al., *Approved CAR-T therapies have reproducible efficacy and safety in clinical practice.* Hum Vaccin Immunother, 2024. **20**(1): p. 2378543.

2. Bisio, M., et al., *Bispecific Antibodies for Lymphoid Malignancy Treatment.* Cancers (Basel), 2024. **17**(1).

3. Caimi, P.F., et al., *Loncastuximab tesirine in relapsed or refractory diffuse large B-cell lymphoma (LOTIS-2): a multicentre, open-label, single-arm, phase 2 trial.* Lancet Oncol, 2021. **22**(6): p. 790-800.

4. Moreau, P., et al., *Teclistamab in Relapsed or Refractory Multiple Myeloma.* N Engl J Med, 2022. **387**(6): p. 495-505.

5. Lesokhin, A.M., et al., *Elranatamab in relapsed or refractory multiple myeloma: phase 2 MagnetisMM-3 trial results.* Nat Med, 2023. **29**(9): p. 2259-2267.

6. Richards, R.M., et al., *NOT-Gated CD93 CAR T Cells Effectively Target AML with Minimized Endothelial Cross-Reactivity.* Blood Cancer Discov, 2021. **2**(6): p. 648-665.

7. Jia, J., et al., *CD93 promotes acute myeloid leukemia development and is a potential therapeutic target.* Exp Cell Res, 2022. **420**(2): p. 113361.

8. Sun, Y., et al., *CD93 blockade promotes effector T-cell infiltration and facilitates adoptive cell therapy in solid tumors.* J Immunother Cancer, 2025. **13**(1).

9. Ali, M., et al., *T cells targeted to TdT kill leukemic lymphoblasts while sparing normal lymphocytes.* Nat Biotechnol, 2022. **40**(4): p. 488-498.
